# Effectiveness and tolerability of brivaracetam in patients with epilepsy stratified by comorbidities and etiology in the real world: 12-month subgroup data from the international EXPERIENCE pooled analysis

**DOI:** 10.1007/s00415-024-12253-z

**Published:** 2024-03-04

**Authors:** Jerzy P. Szaflarski, Hervé Besson, Wendyl D’Souza, Edward Faught, Pavel Klein, Markus Reuber, Felix Rosenow, Javier Salas-Puig, Victor Soto Insuga, Bernhard J. Steinhoff, Adam Strzelczyk, Dimitrios Bourikas, Tony Daniels, Florin Floricel, David Friesen, Cédric Laloyaux, Vicente Villanueva

**Affiliations:** 1 University of Alabama at Birmingham (UAB) Heersink School of Medicine Department of Neurology and UAB Epilepsy Center, Birmingham, AL USA; 2 UCB Pharma, Breda, Netherlands; 3 Department of Medicine, St Vincent’s Hospital Melbourne, The University of Melbourne, Melbourne, VIC Australia; 4 Emory Epilepsy Center, Atlanta, GA USA; 5 Mid-Atlantic Epilepsy and Sleep Center, Bethesda, MD USA; 6 The University of Sheffield, Sheffield, UK; 7 Epilepsy Center Frankfurt Rhine-Main, Department of Neurology, Goethe University Frankfurt, Frankfurt, Germany; 8 LOEWE Center for Personalized Translational Epilepsy Research (CePTER), Goethe University Frankfurt, Frankfurt, Germany; 9 Universitari Vall d’Hebron, Barcelona, Spain; 10 Pediatric Neurology, Hospital Universitario Infantil Niño Jesús, Madrid, Spain; 11 Kork Epilepsy Center, Kehl-Kork and Medical Faculty, University of Freiburg, Freiburg, Germany; 12 UCB Pharma, Alimos, Greece; 13 UCB Pharma, Morrisville, NC USA; 14 UCB Pharma, Monheim am Rhein, Germany; 15 UCB Pharma, Slough, England UK; 16 UCB Pharma, Brussels, Belgium; 17 Refractory Epilepsy Unit, Hospital Universitario y Politécnico La Fe, EpiCARE member, Valencia, Spain

**Keywords:** Brivaracetam, Real world, Comorbidity, Etiology, Effectiveness, Tolerability

## Abstract

**Objective:**

To assess the effectiveness and tolerability of brivaracetam (BRV) in adults with epilepsy by specific comorbidities and epilepsy etiologies.

**Methods:**

EXPERIENCE/EPD332 was a pooled analysis of individual patient records from several non-interventional studies of patients with epilepsy initiating BRV in clinical practice. Outcomes included ≥ 50% reduction from baseline in seizure frequency, seizure freedom (no seizures within prior 3 months), continuous seizure freedom (no seizures since baseline), BRV discontinuation, and treatment-emergent adverse events (TEAEs) at 3, 6, and 12 months. Analyses were performed for all adult patients (≥ 16 years of age) and stratified by comorbidity and by etiology at baseline (patients with cognitive/learning disability [CLD], psychiatric comorbidity, post-stroke epilepsy, brain tumor−related epilepsy [BTRE], and traumatic brain injury−related epilepsy [TBIE]).

**Results:**

At 12 months, ≥ 50% seizure reduction was achieved in 35.6% (*n* = 264), 38.7% (*n* = 310), 41.7% (*n* = 24), 34.1% (*n* = 41), and 50.0% (*n* = 28) of patients with CLD, psychiatric comorbidity, post-stroke epilepsy, BTRE, and TBIE, respectively; and continuous seizure freedom was achieved in 5.7% (*n* = 318), 13.7% (n = 424), 29.4% (n = 34), 11.4% (*n* = 44), and 13.8% (*n* = 29), respectively. During the study follow-up, in patients with CLD, psychiatric comorbidity, post-stroke epilepsy, BTRE, and TBIE, 37.1% (*n* = 403), 30.7% (*n* = 605), 33.3% (*n* = 51), 39.7% (*n* = 68), and 27.1% (*n* = 49) of patients discontinued BRV, respectively; and TEAEs since prior visit at 12 months were reported in 11.3% (*n* = 283), 10.0% (*n* = 410), 16.7% (*n* = 36), 12.5% (*n* = 48), and 3.0% (*n* = 33), respectively.

**Conclusions:**

BRV as prescribed in the real world is effective and well tolerated among patients with CLD, psychiatric comorbidity, post-stroke epilepsy, BTRE, and TBIE.

**Supplementary Information:**

The online version contains supplementary material available at 10.1007/s00415-024-12253-z.

## Introduction

In addition to the number of previous antiseizure medications (ASMs) [1], factors that may affect the response to ASMs in patients with epilepsy include the presence of comorbidities [2] and the underlying epilepsy etiology [3]. Among patients with epilepsy, the prevalence of psychiatric comorbidities ranges from 20 to 50% [4], and 60 to 70% have cognitive impairment [5]. Psychiatric and cognitive comorbidities may be more disabling than the actual seizures themselves [6].

Cognitive comorbidities can adversely affect patient psychosocial functioning, which includes social and emotional competence, well-being, and vocational and educational trajectories [6]. Difficulties with memory, attention, and executive functioning are identified in up to 70% of untreated patients before the onset of seizures or early during diagnosis [6]. Detrimental effects on cognitive function have been reported in some patients with epilepsy in response to treatment with specific ASMs [7]. The risk of developing any psychiatric disorder is two to five times higher in people with epilepsy, and a third of patients with epilepsy have a lifetime history of psychiatric disorders [8]. Poor response to treatment and increased morbidity and mortality have been associated with psychiatric comorbidities [8]. Psychiatric side effects have been reported with some ASMs, which may exacerbate psychiatric disorders in patients with pre-existing psychiatric comorbidities [9, 10]. As such, when selecting an appropriate ASM, the presence of psychiatric comorbidities should be considered [11].

Structural abnormalities leading to epileptic seizures may arise as a result of stroke, brain tumors, and traumatic brain injury [12]. Post-stroke epilepsy occurs following hemorrhagic or ischemic stroke and is the most common cause of acquired epilepsy in older adults [13]. There is limited evidence to support the use of specific ASMs in patients with post-stroke epilepsy, and the choice of ASM is guided by the patients’ comorbidities, sex, age, and co-medications. Brain tumor–related epilepsy (BTRE) is common in patients with cerebral tumors. The risk of BTRE is dependent upon the tumor type, with the incidence ranging from 10 to 15% in patients with brain metastases to > 80% in patients with diffuse low-grade gliomas [14]. Choice of ASM treatment is guided by recommendations for focal epilepsies, and non–enzyme-inducing ASMs are preferred to avoid interference with antineoplastic drugs and support therapies [14]. Post-traumatic epilepsy is defined as epilepsy with recurrent seizures occurring > 7 days after a result of traumatic brain injury [15]. Post-traumatic epilepsy is a common cause of acquired epilepsy and accounts for 10 to 20% of symptomatic epilepsy in the general population [16]. There is little evidence to support the choice of specific ASMs for the symptomatic treatment of seizures in post-traumatic epilepsy [17].

Brivaracetam (BRV) is approved for the treatment of focal-onset (partial-onset) seizures with or without secondary generalization in > 50 countries. The approved age range and adjunctive or monotherapy indication vary by country. EXPERIENCE/EPD332 is an international pooled analysis of individual patient records from multiple independent non-interventional studies in patients with epilepsy initiating BRV in a wide range of geographies (Spain, Germany, Australia, and the United States), clinics, and subgroups [18]. The overall results showed that BRV was effective and well tolerated in patients with epilepsy in routine clinical practice. The large number of patients included in EXPERIENCE (1644 patients ≥ 16 years of age) meant that analyses of specific subgroups were feasible. The objective of these subgroup analyses was to assess the effectiveness and tolerability of BRV in adults with epilepsy by specific comorbidities (cognitive/learning disability [CLD] and psychiatric comorbidity) and epilepsy etiologies (post-stroke epilepsy, BTRE, and traumatic brain injury–related epilepsy [TBIE]).

## Methods

### Study design and patient population

EXPERIENCE/EPD332 was a pooled analysis of patient data from multiple independent, non-interventional, retrospective studies that utilized clinical chart review cohorts of patients who initiated BRV in clinical practice. The primary paper describes the study design in detail [18]. In brief, data were collected from studies that were conducted in Australia, Europe, and the United States that had met the eligibility criteria. In each non-interventional study, patients received BRV as prescribed by their treating physician and according to standard clinical practice in their region. Patient enrollment began with the date of BRV availability in each country; patients must have initiated BRV no earlier than January 2016 and no later than December 2019. Patients had ≥ 6 months of follow-up data from the date of BRV initiation (index date). Each patient had a follow-up period of 12 months after the index date or until one of the following events occurred: BRV discontinuation, death, disenrollment due to any reason, 365 days of follow-up, or end of the study period. Some of the retrospective studies in EXPERIENCE adhered to more specific study protocols, as such data may not adhere exactly to the criteria described above (i.e., follow-up for some patients was > 12 months). For each patient, baseline characteristics were assessed at the index date. Historical variables may have been collected at any point before or at index date.

The terminology used for seizure types is consistent with the terminology used in the original studies, many of which predated the 2017 publication on operational classification of seizure types by the International League Against Epilepsy [19]. EXPERIENCE followed the 2005 Food and Drug Administration’s Guidelines for Good Pharmacoepidemiology Practices (GPP) and the 2008 International Society of Pharmacoepidemiology Guidelines for GPP. Patient data were de-identified before being processed. The EXPERIENCE database consisted of Health Insurance Portability and Accountability Act– and General Data Protection Regulation (GDPR)–compliant anonymized data; as such, no ethics committee approval was required. In order for the Australian and United States cohorts to have their data released and included in the EXPERIENCE database, ethics approval was required. Each non-interventional study that was included in EXPERIENCE received appropriate ethics and/or scientific review board approval as part of the initial study proposal at each institution. For each non-interventional study, appropriate ethics and/or scientific review board approval was obtained as part of the initial study proposal at each institution.

### Outcomes

The following effectiveness outcomes were evaluated at 3, 6, and 12 months after index date: seizure reduction, defined as ≥ 50% reduction from baseline in seizure frequency (patients who had at least one seizure at baseline [modified full analysis set (mFAS)]); seizure freedom, defined as no seizures within 3 months prior to the time point (for some cohorts, seizure freedom was defined as no seizures since the prior visit); continuous seizure freedom, defined as no seizures reported for any time point after baseline; and BRV retention, defined as the number of patients who remained on BRV at each time point. Patients who discontinued BRV were considered to have “no seizure reduction,” and “no seizure freedom” at the time of discontinuation and onward.

The following safety and tolerability outcomes were assessed: BRV discontinuation due to tolerability reasons, defined as the number of patients who discontinued BRV due to tolerability reasons since the prior visit; incidence of treatment-emergent adverse events (TEAEs), defined as TEAEs that occurred since the prior visit; severity of TEAEs; and incidence of psychiatric, cognitive, and behavioral TEAEs.

### Patient subgroups

Outcomes were assessed for all adult patients (≥ 16 years of age), stratified by comorbidity at baseline as documented in the medical records (patients with and without CLD, and patients with and without psychiatric comorbidity) and by etiology at baseline (patients with post-stroke epilepsy and without post-stroke epilepsy, patients with and without BTRE, and patients with and without TBIE). Effectiveness and tolerability outcomes were assessed for patients with psychiatric comorbidity who switched from levetiracetam (LEV) to BRV and in patients who switched from other ASMs (not including LEV) to BRV (patients may have taken LEV historically but stopped LEV treatment long before BRV initiation) at index. The same analysis was undertaken in patients without psychiatric comorbidity. Outcomes were also assessed by etiology and by comorbidity at baseline for patients with focal-onset seizures and a BRV dose of ≤ 200 mg/day used as add-on at index. These analyses represent patients who initiated BRV per either the Australian Product Information [20], the European Summary of Product Characteristics [21], or the US Prescribing Information [22].

### Statistical analyses

Populations analyzed included the full analysis set (FAS), defined as all patients who received at least one dose of BRV and had seizure type and age documented at baseline, and mFAS, defined as all patients in the FAS who had at least one seizure recorded during baseline. Data from the mFAS (based on the estimand at each time point) were used to assess seizure reduction. Data from the FAS (based on the estimand at each time point) were used to assess all other follow-up variables. Assessments of seizure reduction, seizure freedom, and continuous seizure freedom included all seizures recorded during follow-up. Descriptive statistics were used to summarize all variables. With the exception of seizure outcomes (≥ 50% seizure reduction, seizure freedom, and continuous seizure freedom), for which patients with missing data due to BRV discontinuation were deemed to be non-responders for ≥ 50% seizure reduction and not seizure free, no measures were taken to impute or replace missing data. Percentages were based on the number of patients analyzed. Categorical variables were summarized using frequencies and percentages. Analyses were conducted using SAS^®^ (Statistical Analysis System) version 9.4 (SAS Institute, Cary, NC, USA).

## Results

### Subgroup analyses by CLD comorbidity

Subgroup analyses by CLD at baseline included 403 patients with and 1232 patients without CLD (FAS) (Table 1). Patients with CLD were younger than those without CLD (84.9% vs 67.8% were 16–49 years of age). At baseline, the median duration of epilepsy was similar in patients with and without CLD. Patients with CLD had a numerically higher median (25th quartile [Q1], 75th quartile [Q3]) seizure frequency/28 days (7.7 [2.7, 30.0] vs 4.0 [1.0, 12.0]), and numerically higher median (Q1, Q3) number of prior ASMs (any ASM used and stopped before BRV initiation) compared with patients without CLD (7.0 [4.0, 10.0] vs 4.0 [2.0, 7.0]). Neurological and psychiatric comorbidities were more common in patients with than without CLD. A similar percentage of patients with and without CLD switched from LEV to BRV and switched from other ASMs to BRV.

**Table 1:** Baseline demographics and epilepsy characteristics by comorbidity and by etiology (FAS)

Characteristic	CLD comorbidity		Psychiatric comorbidity		Post-stroke epilepsy status		BTRE status		TBIE status	
	With CLD *N* = 403	Without CLD *N* = 1232	With psychiatric comorbidity *N* = 605	Without psychiatric comorbidity *N* = 1011	With post-stroke epilepsy *N* = 51	Without post-stroke epilepsy *N* = 1397	With BTRE *N* = 68	Without BTRE *N* = 1380	With TBIE *N* = 49	Without TBIE *N* = 1399
Age at baseline, *n* (%), years										
16–49	342 (84.9)	835 (67.8)	433 (71.6)	728 (72.0)	17 (33.3)	1028 (73.6)	46 (67.6)	999 (72.4)	29 (59.2)	1016 (72.6)
50–64	46 (11.4)	265 (21.5)	123 (20.3)	185 (18.3)	13 (25.5)	260 (18.7)	15 (22.1)	258 (18.7)	9 (18.4)	264 (18.9)
65–74	13 (3.2)	88 (7.1)	35 (5.8)	66 (6.5)	10 (19.6)	79 (5.7)	5 (7.4)	84 (6.1)	10 (20.4)	79 (5.6)
≥ 75	2 (0.5)	44 (3.6)	14 (2.3)	32 (3.2)	11 (21.6)	30 (2.1)	2 (2.9)	39 (2.8)	1 (2.0)	40 (2.9)
Sex,^a^ *n* (%)										
Male	220 (54.6)	566 (45.9)	274 (45.3)	505 (50.0)	26 (51.0)	661 (47.3)	37 (54.4)	650 (47.1)	34 (69.4)	653 (46.7)
Female	183 (45.4)	665 (54.0)	330 (54.6)	506 (50.0)	25 (49.0)	735 (52.6)	31 (45.6)	729 (52.8)	15 (30.6)	745 (53.3)
Duration of epilepsy, median (Q1, Q3), years	17.0 (5.0, 29.0)^b^	18.0 (9.0, 30.0)^c^	18.0 (8.0, 30.0)^d^	17.0 (8.0, 30.0)^e^	23.5 (3.0, 55.0)^f^	17.0 (8.0, 29.0)^g^	12.0 (2.0, 27.0)	17.8 (8.0, 30.0)^h^	18.0 (9.0, 29.0)	17.0 (7.0, 30.0)^i^
Seizure types at baseline,^j^ *n* (%)										
Focal-onset	360 (89.3)	1148 (93.2)	556 (91.9)	932 (92.2)	51 (100.0)	1274 (91.2)	68 (100.0)	1257 (91.1)	48 (98.0)	1277 (91.3)
Focal-onset with secondary generalization	211 (78.1)^k^	464 (52.8)^l^	279 (66.6)^m^	388 (53.9)^n^	27 (71.1)^o^	595 (64.5)^p^	24 (53.3)^q^	598 (65.4)^r^	28 (75.7)^s^	594 (64.4)^t^
Generalized-onset	42 (10.4)	83 (6.7)	50 (8.3)	76 (7.5)	0	121 (8.7)	0	121 (8.8)	0	121 (8.6)
Unknown-onset	9 (2.2)	4 (0.3)	6 (1.0)	7 (0.7)	0	13 (0.9)	0	13 (0.9)	1 (2.0)	12 (0.9)
Seizure frequency/28 days at index, median (Q1, Q3)	7.7 (2.7, 30.0)^u^	4.0 (1.0, 12.0)^v^	4.0 (1.0, 12.0)^w^	4.0 (1.3, 13.3)^x^	1.0 (0.7, 5.0)^y^	4.0 (1.0, 12.6)^z^	5.3 (1.5, 12.0)	4.0 (1.0, 12.0)^aa^	2.5 (1.0, 8.0)	4.0 (1.0, 12.4)^ab^
Most common etiology (≥ 5% of patients),^j,ac^ *n* (%)										
Malformation of cortical development	71 (17.6)	194 (15.7)	106 (17.5)	157 (15.5)	0	241 (17.3)	1 (1.5)	240 (17.4)	1 (2.0)	240 (17.2)
Genetic	21 (5.2)	68 (5.5)	37 (6.1)	53 (5.2)	0	91 (6.5)	1 (1.5)	90 (6.5)	0	91 (6.5)
Tumor-related	12 (3.0)	71 (5.8)	22 (3.6)	59 (5.8)	1 (2.0)	71 (5.1)	64 (94.1)	8 (0.6)	0	72 (5.1)
Vascular	12 (3.0)	71 (5.8)	29 (4.8)	53 (5.2)	44 (86.3)	26 (1.9)	0	70 (5.1)	0	70 (5.0)
Traumatic	12 (3.0)	45 (3.7)	21 (3.5)	33 (3.3)	0	51 (3.7)	2 (2.9)	49 (3.6)	48 (98.0)	3 (0.2)
Post-infectious	11 (2.7)	28 (2.3)	7 (1.2)	32 (3.2)	3 (5.9)	33 (2.4)	0	36 (2.6)	0	36 (2.6)
Most common comorbid conditions (≥ 10% of patients), *n* (%)										
CLD	403 (100.0)	0	170 (28.2)^ad^	221 (22.0)^ae^	12 (23.5)	354 (25.5)^af^	9 (13.2)	357 (26.0)^ag^	12 (24.5)	354 (25.5)^ah^
Neurological	101 (43.5)^ai^	198 (22.9)^aj^	117 (30.7)^ak^	185 (25.9)^al^	20 (69.0)^am^	263 (30.1)^an^	8 (22.9)^ao^	275 (32.0)^ap^	15 (48.4)^aq^	268 (30.7)^ar^
Psychiatric	170 (43.5)^as^	432 (35.5)^at^	605 (100.0)	0	19 (37.3)	522 (38.0)^au^	20 (30.3)^av^	521 (38.3)^aw^	19 (39.6)^ax^	522 (37.9)^ay^
Cardiovascular disease	22 (6.6)^az^	117 (11.5)^ba^	62 (12.3)^bb^	77 (9.2)^bc^	27 (52.9)	112 (8.6)^bd^	4 (6.2)^be^	135 (10.4)^bf^	8 (17.4)^bg^	131 (10.0)^bh^
Diabetes/endocrine	14 (4.2)^az^	42 (4.1)^bi^	25 (5.0)^bb^	30 (3.6)^bj^	6 (11.8)	50 (3.8)^bk^	3 (4.6)^be^	53 (4.1)^bl^	1 (2.2)^bg^	55 (4.2)^bm^
Cancer	3 (1.5)^bn^	22 (3.1)^bo^	6 (1.9)^bp^	19 (3.2)^bq^	1 (3.4)^am^	24 (2.7)^an^	11 (31.4)^ao^	14 (1.6)^ap^	1 (3.2)^aq^	24 (2.7)^ar^
Prior (lifetime) LEV	188 (61.6)^br^	532 (62.1)^bs^	285 (61.7)^bt^	427 (62.6)^bu^	21 (58.3)^bv^	548 (58.2)^bw^	27 (58.7)^bg^	542 (58.2)^bx^	22 (64.7)^by^	547 (57.9)^bz^
Switched from LEV or other ASMs to BRV, *n* (%)										
Switch from LEV	164 (41.4)^ca^	544 (44.8)^cb^	245 (41.0)^cc^	459 (46.1)^cd^	26 (53.1)^y^	528 (38.3)^ce^	34 (50.7)^cf^	520 (38.2)^cg^	17 (35.4)^ax^	537 (38.9)^ch^
Switch from other ASMs	228 (57.6)^ca^	651 (53.6)^cb^	344 (57.5)^cc^	522 (52.5)^cd^	21 (42.9)^y^	829 (60.2)^ce^	31 (46.3)^cf^	819 (60.2)^cg^	29 (60.4)^ax^	821 (59.5)^ch^
No switch	4 (1.0)^ca^	19 (1.6)^cb^	9 (1.5)^cc^	14 (1.4)^cd^	2 (4.1)^y^	21 (1.5)^ce^	2 (3.0)^cf^	21 (1.5)^cg^	2 (4.2)^ax^	21 (1.5)^ch^
Monotherapy/polytherapy at index, *n* (%)										
Monotherapy^ci^	4 (1.0)	41 (3.3)	20 (3.31)	25 (2.5)	2 (3.9)	39 (2.8)	4 (5.9)	37 (2.7)	4 (8.2)	37 (2.6)
Polytherapy^cj^	399 (99.0)	1191 (96.7)	585 (96.7)	986 (97.5)	49 (96.1)	1358 (97.2)	64 (94.1)	1343 (97.3)	45 (91.8)	1362 (97.4)
Number of prior ASMs at index,^ck^ median (Q1, Q3)	7.0 (4.0, 10.0)^ca^	4.0 (2.0, 7.0)^cl^	5.0 (3.0, 8.0)^cc^	5.0 (2.0, 7.0)^cm^	2.0 (1.0, 4.0)^y^	5.0 (2.0, 8.0)^ch^	3.0 (1.0, 5.0)^cf^	5.0 (2.0, 8.0)^cn^	4.0 (2.0, 7.5)^ax^	5.0 (2.0, 8.0)^co^
0–1, *n* (%)	39 (9.8)^ca^	210 (17.3)^cl^	80 (13.4)^cc^	166 (16.7)^cm^	22 (44.9)^y^	220 (16.0)^ch^	22 (32.8)^cf^	220 (16.2)^cn^	7 (14.6)^ax^	235 (17.0)^co^
2–3, *n* (%)	48 (12.1)^ca^	300 (24.7)^cl^	111 (18.6)^cc^	236 (23.7)^cm^	13 (26.5)^y^	308 (22.3)^ch^	18 (26.9)^cf^	303 (22.3)^cn^	13 (27.1)^ax^	308 (22.3)^co^
4–6, *n* (%)	105 (26.5)^ca^	360 (29.6)^cl^	183 (30.6)^cc^	275 (27.6)^cm^	6 (12.2)^y^	388 (28.1)^ch^	17 (25.4)^cf^	377 (27.7)^cn^	13 (27.1)^ax^	381 (27.6)^co^
≥ 7, *n* (%)	204 (51.5)^ca^	345 (28.4)^cl^	224 (37.5)^cc^	319 (32.0)^cm^	8 (16.3)^y^	463 (33.6)^ch^	10 (14.9)^cf^	461 (33.9)^cn^	15 (31.3)^ax^	456 (33.0)^co^
Number of concomitant maintenance ASMs at index, median (Q1, Q3)	3.0 (1.0, 4.0)	2.0 (1.0, 3.0)	2.0 (1.0, 3.0)	2.0 (1.0, 3.0)	1.0 (1.0, 2.0)	2.0 (1.0, 3.0)	1.0 (1.0, 3.0)	2.0 (1.0, 3.0)	2.0 (1.0, 3.0)	2.0 (1.0, 3.0)

*ASM* antiseizure medication, *BRV* brivaracetam, *BTRE* brain tumor–related epilepsy, *CLD* cognitive/learning disability, *FAS* full analysis set, *LEV* levetiracetam, *Q1* 25th quartile, *Q3* 75th quartile, *TBIE* traumatic brain injury–related epilepsy

^a^One (0.1%) patient in the patients without CLD subgroup, one (0.2%) patient in the patients with psychiatric comorbidity subgroup, one (0.1%) patient in the patients without post-stroke epilepsy subgroup, one (0.1%) patient in the patients without BTRE subgroup, and one (0.1%) patient in the patients without TBIE subgroup reported “other”; ^b^*n* = 393; ^c^*n* = 1204; ^d^*n* = 593; ^e^*n* = 984; ^f^*n* = 50; ^g^*n* = 1358; ^h^*n* = 1340; ^i^*n* = 1359; ^j^Patients could have had more than one response; ^k^*n* = 270; ^l^*n* = 878; ^m^*n* = 419; ^n^*n* = 720; ^o^*n* = 38; ^p^*n* = 922; ^q^*n* = 45; ^r^*n* = 915; ^s^*n* = 37; ^t^*n* = 923; ^u^*n* = 342; ^v^*n* = 1035; ^w^*n* = 519; ^x^*n* = 843; ^y^*n* = 49; ^z^*n* = 1336; ^aa^*n* = 1317; ^ab^*n* = 1336; ^ac^Patients with unknown or other etiology: patients with CLD, 268 (66.5%); patients without CLD, 756 (61.4%); patients with psychiatric comorbidity, 385 (63.6%); patients without psychiatric comorbidity, 627 (62.0%); patients with post-stroke epilepsy, 3 (5.9%); patients without post-stroke epilepsy, 889 (63.6%); patients without BTRE, 892 (64.6%); patients without TBIE, 892 (63.8%); ^ad^*n* = 602; ^ae^*n* = 1006; ^af^*n* = 1388; ^ag^*n* = 1371; ^ah^*n* = 1390; ^ai^*n* = 232; ^aj^*n* = 864; ^ak^*n* = 381; ^al^*n* = 713; ^am^*n* = 29; ^an^*n* = 875; ^ao^*n* = 35; ^ap^*n* = 869; ^aq^*n* = 31; ^ar^*n* = 873; ^as^*n* = 391; ^at^*n* = 1217; ^au^*n* = 1374; ^av^*n* = 66; ^aw^*n* = 1359; ^ax^*n* = 48; ^ay^*n* = 1377; ^az^*n* = 332; ^ba^*n* = 1017; ^bb^*n* = 505; ^bc^*n* = 841; ^bd^*n* = 1307; ^be^*n* = 65; ^bf^*n* = 1293; ^bg^*n* = 46; ^bh^*n* = 1312; ^bi^*n* = 1019; ^bj^*n* = 843; ^bk^*n* = 1309; ^bl^*n* = 1295; ^bm^*n* = 1314; ^bn^*n* = 195; ^bo^*n* = 705; ^bp^*n* = 317; ^bq^*n* = 586; ^br^*n* = 305; ^bs^*n* = 856; ^bt^*n* = 462; ^bu^*n* = 682; ^bv^*n* = 36; ^bw^*n* = 942; ^bx^*n* = 932; ^by^*n* = 34; ^bz^*n* = 944; ^ca^*n* = 396; ^cb^*n* = 1214; ^cc^*n* = 598; ^cd^*n* = 995; ^ce^*n* = 1378; ^cf^*n* = 67; ^cg^*n* = 1360; ^ch^*n* = 1379; ^ci^No concomitant ASM at index; ^cj^Concomitant ASM(s) at index; ^ck^Any ASM used and stopped before BRV initiation; ^cl^*n* = 1215; ^cm^*n* = 996; ^cn^*n* = 1361; ^co^*n* = 1380

The median (Q1, Q3) BRV dose was 100.0 (50.0, 100.0) mg/day in patients with (*n* = 395) and without CLD (*n* = 1211) at index, and was 200.0 (150.0, 200.0) mg/day and 200.0 (100.0, 200.0) mg/day at 12 months in patients with (*n* = 193) and without CLD (*n* = 513), respectively (FAS). The median (Q1, Q3) duration of exposure to BRV was similar in patients with (345.3 [153.3, 396.0] days; *n* = 400), and without CLD (345.0 [124.0, 416.1] days; *n* = 1220). During the whole study follow-up, 37.1% of patients with CLD and 32.6% of patients without CLD discontinued BRV (Table 2). In both subgroups, the two most common reasons for BRV discontinuation (among patients with a documented reason) were “lack of effectiveness” and “tolerability.”

**Table 2:** BRV discontinuation by comorbidity and by etiology (FAS)

Patients, *n* (%)	CLD comorbidity		Psychiatric comorbidity		Post-stroke epilepsy status		BTRE status		TBIE status	
	With CLD *N* = 403	Without CLD *N* = 1232	With psychiatric comorbidity *N* = 605	Without psychiatric comorbidity *N* = 1011	With post-stroke epilepsy *N* = 51	Without post-stroke epilepsy *N* = 1397	With BTRE *N* = 68	Without BTRE *N* = 1380	With TBIE *N* = 49	Without TBIE *N* = 1399
BRV discontinued	149 (37.1)^a^	400 (32.6)^b^	185 (30.7)^c^	357 (35.4)^d^	17 (33.3)	471 (33.8)^e^	27 (39.7)	461 (33.5)^f^	13 (27.1)^g^	475 (34.1)^h^
Reason for BRV discontinuation^i^										
Lack of effectiveness	81 (55.1)^j^	161 (40.7)^k^	79 (43.4)^l^	159 (44.9)^m^	3 (17.6)^n^	224 (48.2)^o^	8 (29.6)^p^	219 (48.1)^q^	7 (53.8)^r^	220 (46.9)^s^
Tolerability	40 (27.2)^j^	150 (37.9)^k^	66 (36.3)^l^	122 (34.5)^m^	6 (35.3)^n^	153 (32.9)^o^	11 (40.7)^p^	148 (32.5)^q^	2 (15.4)^r^	157 (33.5)^s^
Lack of effectiveness and tolerability	26 (17.7)^j^	47 (11.9)^k^	31 (17.0)^l^	41 (11.6)^m^	6 (35.3)^n^	53 (11.4)^o^	4 (14.8)^p^	55 (12.1)^q^	0	59 (12.6)^s^
Other	7 (4.8)^j^	55 (13.9)^k^	13 (7.1)^l^	49 (13.8)^m^	2 (11.8)^n^	59 (12.7)^o^	5 (18.5)^p^	56 (12.3)^q^	3 (23.1)^r^	58 (12.4)^s^
Cost	4 (2.7)^j^	8 (2.0)^k^	3 (1.6)^l^	9 (2.5)^m^	0	12 (2.6)^o^	1 (3.7)^p^	11 (2.4)^q^	1 (7.7)^r^	11 (2.3)^s^
BRV availability	1 (0.7)^j^	1 (0.3)^k^	0	2 (0.6)^m^	0	2 (0.4)^o^	0	2 (0.4)^q^	0	2 (0.4)^s^
BRV discontinued due to tolerability										
In the first 3 months	28 (19.2)^t^	69 (17.6)^u^	41 (22.9)^v^	56 (15.9)^w^	6 (37.5)^x^	88 (19.0)^y^	8 (29.6)^p^	86 (19.0)^z^	2 (15.4)^r^	92 (19.7)^aa^
Between 3 and 6 months	17 (11.6)^t^	69 (17.6)^u^	34 (19.0)^v^	49 (13.9)^w^	3 (18.8)^x^	60 (13.0)^y^	4 (14.8)^p^	59 (13.1)^z^	0	63 (13.5)^aa^
Between 6 and 12 months	14 (9.6)^t^	38 (9.7)^u^	14 (7.8)^v^	38 (10.8)^w^	3 (18.8)^x^	35 (7.6)^y^	2 (7.4)^p^	36 (8.0)^z^	0	38 (8.2)^aa^

*BRV* brivaracetam, *BTRE* brain tumor–related epilepsy, *CLD* cognitive/learning disability, *FAS* full analysis set, *TBIE* traumatic brain injury–related epilepsy

^a^*n* = 402; ^b^*n* = 1228; ^c^*n* = 603; ^d^*n* = 1008; ^e^*n* = 1392; ^f^*n* = 1375; ^g^*n* = 48; ^h^*n* = 1395; ^i^Reasons for BRV discontinuation were not mutually exclusive; ^j^*n* = 147; ^k^*n* = 396; ^l^*n* = 182; ^m^*n* = 354; ^n^*n* = 17; ^o^*n* = 465; ^p^*n* = 27; ^q^*n* = 455; ^r^*n* = 13; ^s^*n* = 469; ^t^*n* = 146; ^u^*n* = 392; ^v^*n* = 179; ^w^*n* = 352; ^x^*n* = 16; ^y^*n* = 463; ^z^*n* = 452; ^aa^*n* = 466

**Fig. 1:**
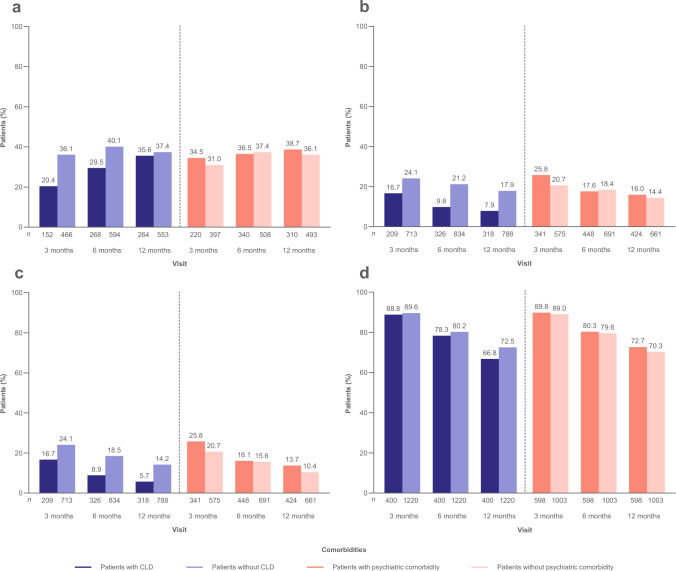
Analyses of effectiveness by comorbidity (patients with/without CLD and patients with/without psychiatric comorbidity) at baseline: **a**  ≥ 50% seizure reduction (mFAS), **b** seizure freedom (FAS), **c** continuous seizure freedom (FAS), and **d** BRV retention (FAS). *n* represents the number of patients with data for the reported variable at each visit. Patients with missing data were excluded from all seizure analyses. Patients with missing data after BRV discontinuation were considered non-responders and not seizure free. *BRV* brivaracetam, *CLD* cognitive/learning disability, *FAS* full analysis set, *mFAS* modified full analysis set

At 12 months, ≥ 50% seizure reduction was achieved in 35.6% and 37.4% of patients with and without CLD, respectively (mFAS) (Fig. 1a); seizure freedom was achieved in 7.9% and 17.9% (FAS) (Fig. 1b); continuous seizure freedom was achieved in 5.7% and 14.2% (Fig. 1c); and BRV retention was achieved in 66.8% and 72.5% (Fig. 1d).

Both subgroups of patients had similar incidences of TEAEs at 3, 6, and 12 months (FAS) (Table 3). Incidences of cognitive TEAEs were low at 3, 6, and 12 months. The incidences of psychiatric and behavioral TEAEs were also low in patients with and without CLD at all time points.

**Table 3:** TEAEs since prior visit, by comorbidity (FAS)

Patients, *n* (%)	3 months				6 months				12 months			
	CLD comorbidity		Psychiatric comorbidity		CLD comorbidity		Psychiatric comorbidity		CLD comorbidity		Psychiatric comorbidity	
	With CLD *n* = 370	Without CLD *n* = 1163	With psychiatric comorbidity *n* = 557	Without psychiatric comorbidity *n* = 957	With CLD *n* = 323	Without CLD *n* = 1045	With psychiatric comorbidity *n* = 478	Without psychiatric comorbidity *n* = 875	With CLD *n* = 283	Without CLD *n* = 942	With psychiatric comorbidity *n* = 410	Without psychiatric comorbidity *n* = 803
Any TEAEs	84 (22.7)	308 (26.5)	151 (27.1)	236 (24.7)	50 (15.5)	144 (13.8)	67 (14.0)	122 (13.9)	32 (11.3)	82 (8.7)	41 (10.0)	71 (8.8)
Severity of TEAEs^a,b^												
Mild	31 (51.7)^c^	107 (49.1)^d^	57 (47.9)^e^	77 (50.3)^f^	13 (39.4)^g^	56 (59.6)^h^	32 (59.3)^i^	32 (47.1)^j^	8 (33.3)^k^	27 (50.9)^l^	19 (57.6)^g^	15 (35.7)^m^
Moderate	20 (33.3)^c^	83 (38.1)^d^	46 (38.7)^e^	56 (36.6)^f^	16 (48.5)^g^	33 (35.1)^h^	18 (33.3)^i^	31 (45.6)^j^	13 (54.2)^k^	24 (45.3)^l^	12 (36.4)^g^	24 (57.1)^m^
Severe	9 (15.0)^c^	27 (12.4)^d^	15 (12.6)^e^	20 (13.1)^f^	4 (12.1)^g^	5 (5.3)^h^	4 (7.4)^i^	5 (7.4)^j^	3 (12.5)^k^	2 (3.8)^l^	2 (6.1)^g^	3 (7.1)^m^
Life-threatening	0	1 (0.5)^d,n^	1 (0.8)^e,n^	0	0	0	0	0	0	0	0	0
Psychiatric TEAEs^o^	20 (5.4)	77 (6.6)	38 (6.8)	55 (5.7)	11 (3.4)	24 (2.3)	10 (2.1)	23 (2.6)	10 (3.5)	21 (2.2)	11 (2.7)	20 (2.5)
Cognitive TEAEs	8 (2.2)	33 (2.8)	15 (2.7)	24 (2.5)	6 (1.9)	11 (1.1)	5 (1.0)	13 (1.5)	3 (1.1)	8 (0.8)	5 (1.2)	7 (0.9)
Behavioral TEAEs	28 (7.6)	52 (4.5)	27 (4.8)	50 (5.2)	16 (5.0)	22 (2.1)	14 (2.9)	21 (2.4)	9 (3.2)	9 (1.0)	7 (1.7)	10 (1.2)
TEAEs^p,q^ reported by ≥ 3% of patients at any time point												
Irritability	21 (5.7)	41 (3.5)	23 (4.1)	38 (4.0)	11 (3.4)	18 (1.7)	12 (2.5)	16 (1.8)	4 (1.4)	7 (0.7)	5 (1.2)	6 (0.7)
Somnolence	13 (3.5)	47 (4.0)	22 (3.9)	38 (4.0)	9 (2.8)	26 (2.5)	14 (2.9)	20 (2.3)	6 (2.1)	21 (2.2)	11 (2.7)	15 (1.9)
Fatigue	6 (1.6)	58 (5.0)	24 (4.3)	39 (4.1)	1 (0.3)	19 (1.8)	9 (1.9)	9 (1.0)	2 (0.7)	8 (0.8)	6 (1.5)	3 (0.4)
Dizziness	8 (2.2)	55 (4.7)	29 (5.2)	35 (3.7)	1 (0.3)	15 (1.4)	6 (1.3)	10 (1.1)	0	11 (1.2)	7 (1.7)	4 (0.5)

*AE* adverse event, *CLD* cognitive/learning disability, *FAS* full analysis set, *TEAE* treatment-emergent adverse event

^a^Patients with reported severity; ^b^Excluding patients who had an AE that was not further described; ^c^*n* = 60; ^d^*n* = 218; ^e^*n* = 119; ^f^*n* = 153; ^g^*n* = 33; ^h^*n* = 94; ^i^*n* = 54; ^j^*n* = 68; ^k^*n* = 24; ^l^*n* = 53; ^m^*n* = 42; ^n^One TEAE of suicide was documented as life-threatening; ^o^Behavioral TEAEs that fulfilled the criteria for psychiatric TEAEs were included in the psychiatric TEAEs category; ^p^Medical Dictionary for Regulatory Activities version 24.1; ^q^Patients with recorded AE that was not further described at 3 months/6 months/12 months: patients with CLD, 8 (2.2%)/14 (4.3%)/7 (2.8%); patients without CLD, 21 (1.8%)/26 (2.5%)/10 (1.1%); patients with psychiatric comorbidity, 17 (3.1%)/10 (2.1%)/1 (0.2%); patients without psychiatric comorbidity, 12 (1.3%)/30 (3.4%)/16 (2.0%)

### Subgroup analyses by psychiatric comorbidity

Subgroup analyses by psychiatric comorbidity at baseline included 605 patients with and 1011 patients without psychiatric comorbidity (FAS). Baseline characteristics were generally similar among patients with and without psychiatric comorbidity (Table 1). CLD comorbidity was more common in patients with than without psychiatric comorbidity. Prior (lifetime) LEV use was reported in 61.7% of patients with and 62.6% without psychiatric comorbidity. A numerically lower percentage of patients with than without psychiatric comorbidity switched from LEV to BRV (41.0% vs 46.1%), and a numerically higher percentage switched from other ASMs to BRV (57.5% vs 52.5%).

In both subgroups, median (Q1, Q3) BRV dose was 100.0 (50.0, 100.0) mg/day at index (with psychiatric comorbidity, *n* = 597; without psychiatric comorbidity, *n* = 996), and 200.0 (100.0, 200.0) mg/day at 12 months (with psychiatric comorbidity, *n* = 277; without psychiatric comorbidity, *n* = 415) (FAS). The median (Q1, Q3) duration of exposure to BRV was similar in patients with (341.5 [119.0, 398.1] days; *n* = 598) and without psychiatric comorbidity (349.0 [143.7, 420.0] days; *n* = 1003). During the whole study follow-up, 30.7% of patients with psychiatric comorbidity and 35.4% of patients without psychiatric comorbidity discontinued BRV (Table 2). In both subgroups, the most common reasons for BRV discontinuation (among patients with a documented reason) were “lack of effectiveness” and “tolerability.”

At 12 months, ≥ 50% seizure reduction was achieved in 38.7% and 36.1% of patients with and without psychiatric comorbidity, respectively (mFAS) (Fig. 1a); seizure freedom was achieved in 16.0% and 14.4% (FAS) (Fig. 1b); continuous seizure freedom was achieved in 13.7% and 10.4% (Fig. 1c); and BRV retention was achieved in 72.7% and 70.3% (Fig. 1d).

In patients with psychiatric comorbidity who switched from LEV to BRV and who switched from other ASMs to BRV, at 12 months, ≥ 50% seizure reduction was achieved in 38.3% and 38.7% of patients, respectively (mFAS) (Supplementary Fig. S1a); seizure freedom was achieved in 13.9% and 16.2% (FAS) (Supplementary Fig. S1b); continuous seizure freedom was achieved in 10.6% and 15.0% (Supplementary Fig. S1c); and BRV retention was achieved in 73.3% and 71.4% (Supplementary Fig. S1d).

In patients with and without psychiatric comorbidity, the incidences of TEAEs were similar at 3, 6, and 12 months (FAS) (Table 3). Incidences of psychiatric TEAEs were similar in patients with and without psychiatric comorbidity at 3, 6, and 12 months. Incidences of cognitive and behavioral TEAEs were low in both patient subgroups. At 3, 6, and 12 months, patients with psychiatric comorbidity who switched from LEV to BRV had similar incidences of psychiatric, cognitive, and behavioral TEAEs to patients with psychiatric comorbidity who switched from other ASMs to BRV (Supplementary Table S1). Similar incidences of psychiatric, cognitive, and behavioral TEAEs were also observed in patients without psychiatric comorbidity who switched from LEV to BRV compared with patients who switched from other ASMs to BRV.

### Subgroup analyses by post-stroke epilepsy status

Subgroup analyses by post-stroke epilepsy status at baseline included 51 patients with and 1397 patients without post-stroke epilepsy (FAS) (Table 1). At baseline, patients with post-stroke epilepsy were older (41.2% [*n* = 21] vs 7.8% [*n* = 109] were ≥ 65 years of age), had a longer median duration of epilepsy, more commonly had focal-onset seizures, had a lower median (Q1, Q3) seizure frequency/28 days (1.0 [0.7, 5.0] vs 4.0 [1.0, 12.6]), and a lower median (Q1, Q3) number of prior ASMs (2.0 [1.0, 4.0] vs 5.0 [2.0, 8.0]) compared with those without post-stroke epilepsy. Neurological conditions, cardiovascular disease, and diabetes/endocrine conditions were more common in patients with (69.0%, 52.9%, and 11.8%, respectively) than without post-stroke epilepsy (30.1%, 8.6%, and 3.8%, respectively). A numerically higher percentage of patients with than without post-stroke epilepsy switched from LEV to BRV, and a numerically lower percentage switched from other ASMs to BRV.

The median (Q1, Q3) BRV dose was 50.0 (50.0, 100.0) mg/day (*n* = 51) and 100.0 (50.0, 100.0) mg/day (*n* = 1377) at index in patients with and without post-stroke epilepsy, respectively, and 100.0 (85.5, 150.0) mg/day (*n* = 20) and 200.0 (100.0, 200.0) mg/day (*n* = 555) at 12 months (FAS). The median (Q1, Q3) duration of exposure to BRV was similar in patients with (350.1 [91.5, 405.1] days; *n* = 50) and without post-stroke epilepsy (343.0 [123.9, 414.3] days; *n* = 1385).

During the whole study follow-up, 33.3% of patients with post-stroke epilepsy and 33.8% of patients without post-stroke epilepsy discontinued BRV (Table 2). The most common reasons for BRV discontinuation (among patients with a documented reason) were “tolerability” and “lack of effectiveness and tolerability” in patients with post-stroke epilepsy and “lack of effectiveness” and “tolerability” in patients without post-stroke epilepsy.

**Fig. 2:**
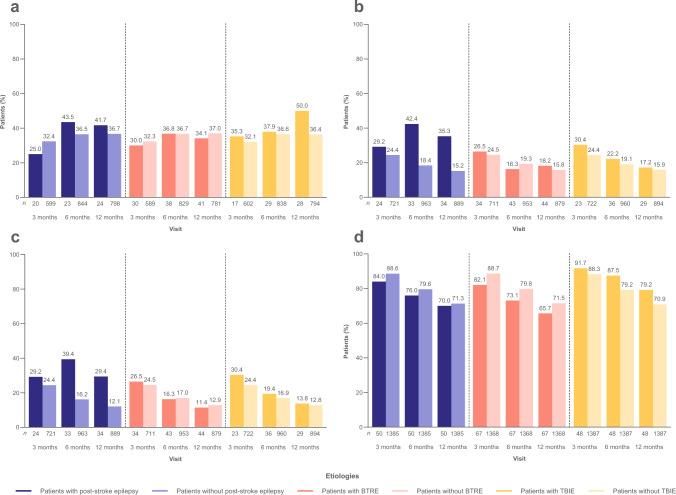
Analyses of effectiveness by etiology (patients with/without post-stroke epilepsy, patients with/without BTRE, and patients with/without TBIE) at baseline: **a ** ≥ 50% seizure reduction (mFAS), **b** seizure freedom (FAS), **c** continuous seizure freedom (FAS), and **d** BRV retention (FAS). *n* represents the number of patients with data for the reported variable at each visit. Patients with missing data were excluded from all seizure analyses. Patients with missing data after BRV discontinuation were considered non-responders and not seizure free. *BRV* brivaracetam, *BTRE* brain tumor–related epilepsy, *FAS* full analysis set, *mFAS* modified full analysis set, *TBIE* traumatic brain injury–related epilepsy

At 12 months, ≥ 50% seizure reduction was achieved in 41.7% and 36.7% of patients with and without post-stroke epilepsy, respectively (mFAS) (Fig. 2a); seizure freedom was achieved in 35.3% and 15.2% (FAS) (Fig. 2b); continuous seizure freedom was achieved in 29.4% and 12.1% (Fig. 2c); and BRV retention was 70.0% and 71.3% (Fig. 2d).

The incidences of TEAEs in patients with and without post-stroke epilepsy were 34.1% and 24.0%, respectively, at 3 months; 25.6% and 13.3% at 6 months; and 16.7% and 7.9% at 12 months (FAS) (Table 4). At 3 months, the incidences of psychiatric TEAEs were 13.6% and 5.8% in patients with and without post-stroke epilepsy, respectively; 2.6% and 2.3% at 6 months; and 2.8% and 2.4% at 12 months. Incidences of cognitive and behavioral TEAEs at 3, 6, and 12 months were low in patients with and without post-stroke epilepsy.

**Table 4:** TEAEs since prior visit, by etiology (FAS)

Patients, *n* (%)	3 months						6 months						12 months					
	Post-stroke epilepsy status		BTRE status		TBIE status		Post-stroke epilepsy status		BTRE status		TBIE status		Post-stroke epilepsy status		BTRE status		TBIE status	
	With post-stroke epilepsy *n* = 44	Without post-stroke epilepsy *n* = 1302	With BTRE *n* = 65	Without BTRE *n* = 1281	With TBIE *n* = 42	Without TBIE *n* = 1304	With post-stroke epilepsy *n* = 39	Without post-stroke epilepsy *n* = 1177	With BTRE *n* = 53	Without BTRE *n* = 1163	With TBIE *n* = 38	Without TBIE *n* = 1178	With post-stroke epilepsy *n* = 36	Without post-stroke epilepsy *n* = 1057	With BTRE *n* = 48	Without BTRE *n* = 1045	With TBIE *n* = 33	Without TBIE *n* = 1060
Any TEAEs	15 (34.1)	313 (24.0)	18 (27.7)	310 (24.2)	8 (19.0)	320 (24.5)	10 (25.6)	157 (13.3)	7 (13.2)	160 (13.8)	2 (5.3)	165 (14.0)	6 (16.7)	84 (7.9)	6 (12.5)	84 (8.0)	1 (3.0)	89 (8.4)
Severity of TEAEs^a,b^																		
Mild	7 (77.8)^c^	110 (53.7)^d^	8 (61.5)^e^	109 (54.2)^f^	3 (60.0)^g^	114 (54.5)^h^	5 (71.4)^i^	55 (57.9)^j^	3 (100.0)^k^	57 (57.6)^l^	2 (100.0)^m^	58 (58.0)^n^	4 (100.0)^o^	23 (42.6)^p^	1 (50.0)^m^	26 (46.4)^q^	1 (100.0)^r^	26 (45.6)^s^
Moderate	2 (22.2)^c^	75 (36.6)^d^	4 (30.8)^e^	73 (36.3)^f^	2 (40.0)^g^	75 (35.9)^h^	2 (28.6)^i^	35 (36.8)^j^	0	37 (37.4)^l^	0	37 (37.0)^n^	0	28 (51.9)^p^	1 (50.0)^m^	27 (48.2)^q^	0	28 (49.1)^s^
Severe	0	19 (9.3)^d^	1 (7.7)^e^	18 (9.0)^f^	0	19 (9.1)^h^	0	5 (5.3)^j^	0	5 (5.1)^l^	0	5 (5.0)	0	3 (5.6)^p^	0	3 (5.4)^q^	0	3 (5.3)^s^
Life-threatening	0	1 (0.5)^d, t^	0	1 (0.5)^f, t^	0	1 (0.5)^h, t^	0	0	0	0	0	0	0	0	0	0	0	0
Psychiatric TEAEs^u^	6 (13.6)	76 (5.8)	5 (7.7)	77 (6.0)	0	82 (6.3)	1 (2.6)	27 (2.3)	3 (5.7)	25 (2.1)	0	28 (2.4)	1 (2.8)	25 (2.4)	2 (4.2)	24 (2.3)	0	26 (2.5)
Cognitive TEAEs	1 (2.3)	40 (3.1)	2 (3.1)	39 (3.0)	1 (2.4)	40 (3.1)	0	18 (1.5)	1 (1.9)	17 (1.5)	0	18 (1.5)	0	12 (1.1)	1 (2.1)	11 (1.1)	0	12 (1.1)
Behavioral TEAEs	4 (9.1)	60 (4.6)	3 (4.6)	61 (4.8)	2 (4.8)	62 (4.8)	1 (2.6)	29 (2.5)	1 (1.9)	29 (2.5)	0	30 (2.5)	0	12 (1.1)	1 (2.1)	11 (1.1)	0	12 (1.1)
TEAEs^v,w^ reported by ≥ 3% of patients at any time point																		
Depression	3 (6.8)	32 (2.5)	3 (4.6)	32 (2.5)	0	35 (2.7)	0	3 (0.3)	0	3 (0.3)	0	3 (0.3)	1 (2.8)	3 (0.3)	0	4 (0.4)	0	4 (0.4)
Aggression	2 (4.5)	18 (1.4)	2 (3.1)	18 (1.4)	0	20 (1.5)	0	11 (0.9)	1 (1.9)	10 (0.9)	0	11 (0.9)	0	6 (0.6)	0	6 (0.6)	0	6 (0.6)
Fatigue	2 (4.5)	58 (4.5)	5 (7.7)	55 (4.3)	2 (4.8)	58 (4.4)	1 (2.6)	15 (1.3)	1 (1.9)	15 (1.3)	0	16 (1.4)	1 (2.8)	8 (0.8)	1 (2.1)	8 (0.8)	0	9 (0.8)
Somnolence	2 (4.5)	38 (2.9)	1 (1.5)	39 (3.0)	0	40 (3.1)	3 (7.7)	23 (2.0)	0	26 (2.2)	0	26 (2.2)	1 (2.8)	15 (1.4)	0	16 (1.5)	0	16 (1.5)
Irritability	1 (2.3)	45 (3.5)	2 (3.1)	44 (3.4)	2 (4.8)	44 (3.4)	1 (2.6)	20 (1.7)	0	21 (1.8)	0	21 (1.8)	0	5 (0.5)	0	5 (0.5)	0	5 (0.5)
Dizziness	0	48 (3.7)	2 (3.1)	46 (3.6)	1 (2.4)	47 (3.6)	0	12 (1.0)	1 (1.9)	11 (0.9)	0	12 (1.0)	0	6 (0.6)	0	6 (0.6)	0	6 (0.6)

*AE* adverse event, *BTRE* brain tumor–related epilepsy, *FAS* full analysis set, *TBIE* traumatic brain injury–related epilepsy, *TEAE* treatment-emergent adverse event

^a^Patients with reported severity; ^b^Excluding patients who had an AE that was not further described; ^c^*n* = 9; ^d^*n* = 205; ^e^*n* = 13; ^f^*n* = 201; ^g^*n* = 5; ^h^*n* = 209; ^i^*n* = 7; ^j^*n* = 95; ^k^*n* = 3; ^l^*n* = 99; ^m^*n* = 2; ^n^*n* = 100; ^o^*n* = 4; ^p^*n* = 54; ^q^*n* = 56; ^r^*n* = 1; ^s^*n* = 57; ^t^One TEAE of suicide was documented as life-threatening; ^u^Behavioral TEAEs that fulfilled the criteria for psychiatric TEAEs were included in the psychiatric TEAEs category; ^v^Medical Dictionary for Regulatory Activities version 24.1; ^w^Patients with recorded AE that was not further described at 3 months/6 months/12 months: patients with post-stroke epilepsy, 3 (6.8%)/2 (5.1%)/1 (2.8%); patients without post-stroke epilepsy, 26 (2.0%)/38 (3.2%)/16 (1.5%); patients with BTRE, 1 (1.5%)/1 (1.9%)/2 (4.2%); patients without BTRE, 28 (2.2%)/39 (3.4%)/15 (1.4%); patients with TBIE 2, (4.8%)/1 (2.6%)/1 (3.0%), patients without TBIE, 27 (2.1%)/39 (3.3%)/16 (1.5%)

### Subgroup analyses by BTRE status

Subgroup analyses by BTRE status at baseline included 68 patients with and 1380 patients without BTRE (FAS) (Table 1). Patients with BTRE had a shorter median (Q1, Q3) duration of epilepsy (12.0 [2.0, 27.0] vs 17.8 [8.0, 30.0]), more commonly had focal-onset seizures, and had a numerically lower median (Q1, Q3) number of prior ASMs compared with patients without BTRE (3.0 [1.0, 5.0] vs 5.0 [2.0, 8.0]). The median (Q1, Q3) seizure frequency/28 days was 5.3 (1.5, 12.0) and 4.0 (1.0, 12.0) in patients with and without BTRE, respectively. Cancer comorbidity, CLD, and neurological conditions were more common in patients with BTRE than those without BTRE, and psychiatric conditions were less common. A numerically higher percentage of patients with than without BTRE switched from LEV to BRV (50.7% vs 38.2%), and a lower percentage switched from other ASMs (46.3% vs 60.2%).

The median (Q1, Q3) BRV dose was 50.0 (50.0, 100.0) mg/day (*n* = 67) and 100.0 (50.0, 100.0) mg/day (*n* = 1361) at index in patients with and without BTRE, respectively, and 200.0 (100.0, 200.0) mg/day at 12 months in both subgroups (with BTRE, *n* = 25; without BTRE, *n* = 550; FAS). The median (Q1, Q3) duration of exposure to BRV was similar in patients with (349.7 [105.0, 410.9] days; *n* = 67) and without BTRE (343.0 [122.0, 413.3] days; *n* = 1368).

During the whole study follow-up, 39.7% of patients with BTRE and 33.5% of patients without BTRE discontinued BRV (Table 2). The most common reasons for BRV discontinuation in both subgroups (among patients with a documented reason) were “lack of effectiveness” (with BTRE, 29.6%; without BTRE, 48.1%) and “tolerability” (40.7%; 32.5%).

At 12 months, ≥ 50% seizure reduction was achieved in 34.1% and 37.0% of patients with and without BTRE, respectively (mFAS) (Fig. 2a); seizure freedom was achieved in 18.2% and 15.8% (FAS) (Fig. 2b); continuous seizure freedom was achieved in 11.4% and 12.9% (Fig. 2c); and BRV retention was achieved in 65.7% and 71.5% (Fig. 2d).

The incidence of TEAEs at 3, 6, and 12 months was similar among patients with and without BTRE (FAS) (Table 4). Incidences of psychiatric, cognitive, and behavioral TEAEs were low in both subgroups of patients.

### Subgroup analyses by TBIE status

Subgroup analyses by TBIE status at baseline included 49 patients with and 1399 patients without TBIE (FAS) (Table 1). Patients with TBIE were older than patients without TBIE, and a higher percentage were male. At baseline, patients with and without TBIE had a similar median duration of epilepsy, and patients with TBIE had a numerically lower median seizure frequency/28 days. The median number of prior ASMs was similar in patients with and without TBIE. Neurological conditions and cardiovascular disease were more common in patients with than without TBIE. A similar percentage of patients with and without TBIE switched from LEV to BRV and switched from other ASMs to BRV.

The median (Q1, Q3) BRV dose was 100.0 (50.0, 100.0) mg/day at index in both subgroups (with TBIE, *n* = 47; without TBIE, *n* = 1381); and 175.0 (100.0, 200.0) mg/day (*n* = 19), and 200.0 (100.0, 200.0) mg/day (*n* = 556) at 12 months in patients with and without TBIE, respectively (FAS). The median (Q1, Q3) duration of exposure to BRV was 352.9 (176.0, 441.0) days in patients with TBIE (*n* = 48), and 343.0 (122.0, 413.0) days in patients without TBIE (*n* = 1387).

During the whole study follow-up, 27.1% of patients with TBIE and 34.1% of patients without TBIE discontinued BRV (Table 2). The most common reason for BRV discontinuation in both subgroups (among patients with a documented reason) was “lack of effectiveness.” A numerically higher percentage of patients with TBIE than without TBIE discontinued BRV due to “lack of effectiveness” (53.8% vs 46.9%), “other” reasons (23.1% vs 12.4%), and cost (7.7% vs 2.3%); and a numerically lower percentage discontinued due to “tolerability” (15.4% vs 33.5%), and “lack of effectiveness and tolerability” (0% vs 12.6%).

At 12 months, ≥ 50% seizure reduction was achieved in 50.0% and 36.4% of patients with and without TBIE, respectively (mFAS) (Fig. 2a); seizure freedom was achieved in 17.2% and 15.9% (FAS) (Fig. 2b); continuous seizure freedom was achieved in 13.8% and 12.8% (Fig. 2c); and BRV retention was achieved in 79.2% and 70.9% (Fig. 2d).

The incidences of TEAEs in patients with and without TBIE were 19.0% and 24.5%, respectively, at 3 months; 5.3% and 14.0% at 6 months; and 3.0% and 8.4% at 12 months (FAS) (Table 4). No psychiatric TEAEs were reported at 3, 6, or 12 months in patients with TBIE, and no cognitive or behavioral TEAES were reported at 6 or 12 months. Among patients without TBIE, incidences of psychiatric, cognitive, and behavioral TEAEs were low.

### Patients with focal-onset seizures who were on a BRV dose of ≤ 200 mg/day used as add-on at index

When analyses were restricted to patients with focal-onset seizures who had a BRV dose of ≤ 200 mg/day used as add-on at index, the effectiveness and tolerability results were similar to those observed in the wider subgroups of patients with and without each comorbidity (CLD and psychiatric comorbidity) or etiology (post-stroke epilepsy, BTRE, and TBIE) (Supplementary Appendix S1, Supplementary Tables S2–S4, Supplementary Figs. S2–S3).

## Discussion

In patients with epilepsy, the presence of comorbidities and underlying epilepsy etiology may affect the response to ASMs and should be considered when selecting the most appropriate treatment. Some patients with epilepsy have reported negative cognitive effects in response to treatment with specific ASMs [7]. Poor response to treatment has been associated with psychiatric comorbidities [8], and some ASMs are reported to cause psychiatric side effects [9, 10]. There is limited evidence to support the choice of ASMs for specific etiologies such as post-stroke epilepsy and TBIE; however, for BTRE, the use of non–enzyme-inducing ASMs is recommended [23].

The EXPERIENCE analysis provides a large amount of data for the evaluation of 12-month BRV effectiveness and tolerability among patient subgroups [18]. Patients included in EXPERIENCE were drug resistant, as evidenced by their baseline characteristics. In these subgroup analyses, BRV was effective and well tolerated in patients with CLD and psychiatric comorbidity, and in patients with various structural epilepsy etiologies (post-stroke epilepsy, BTRE, and TBIE).

### Subgroup analyses by CLD comorbidity

A prospective observational study of adults with drug-resistant epilepsy in the United Kingdom showed similar ≥ 50% seizure reduction between patients with and without intellectual disability (37.0% vs 32.0%) [24]. In EXPERIENCE, patients with CLD tended to have lower ≥ 50% seizure reduction at 3 and 6 months, and lower seizure freedom and continuous seizure freedom at 3, 6, and 12 months, compared with patients without CLD. This is likely due to the more drug-resistant population. Patients with CLD had a high median number of prior ASMs (7.0 vs 4.0 in those without CLD) and a higher median seizure frequency/28 days at index (7.7 vs 4.0). Retention on BRV was high in patients with and without CLD, indicating they were generally satisfied with their treatment. In line with these results, the UK study showed similar retention on BRV in patients with and without intellectual disability (66.0% vs 62.0%) [24].

A retrospective observational study conducted in Germany showed BRV to be effective in drug-resistant patients with intellectual disability [25]. A ≥ 50% seizure reduction was reported by 19% of patients after 12 months, lower than that reported in EXPERIENCE (35.6%). These differences may reflect differences in study design; the Germany retrospective study was a single-center study with only 33 patients.

In EXPERIENCE, “lack of effectiveness” was the most common reason for discontinuation among patients with CLD. BRV did not appear to exacerbate CLD, as shown by the low incidences of cognitive TEAEs among patients with these comorbidities. A favorable cognitive profile for BRV was shown in a randomized, placebo-controlled, double-blind, four-way crossover study in 16 healthy volunteers [26]. The effects of BRV on electrophysiologic, cognitive, and subjective measures were comparable with those of LEV (known to have favorable cognitive profile) and placebo [26].

### Subgroup analyses by psychiatric comorbidity

Analyses in patients with and without psychiatric comorbidity showed that ≥ 50% seizure reduction, seizure freedom, continuous seizure freedom, and retention on BRV were generally similar in both subgroups. In line with these results, a prospective observational study of adult patients with drug-resistant epilepsy treated with adjunctive BRV for a mean of 11 months in the United Kingdom showed similar ≥ 50% seizure reduction and retention on BRV between patients with and without psychiatric or behavioral comorbidities (29.0% vs 39.0%, and 60.0% vs 67.0%, respectively) [24].

Psychiatric comorbidities are associated with an increased risk of cognitive and psychiatric side effects [8]. A retrospective study of 1058 patients with uncontrolled seizures showed that those with a history of psychiatric comorbidity were more likely to discontinue a newly administered ASM due to psychiatric issues than those with no previous psychiatric comorbidity [27]. In EXPERIENCE, discontinuations of BRV due to tolerability reasons were similar in patients with and without psychiatric comorbidity. Few patients reported psychiatric or cognitive TEAEs, suggesting that BRV treatment did not exacerbate pre-existing psychiatric comorbidity. This finding is supported by data from the UK observational study, which showed similar tolerability in patients with and without pre-existing psychiatric or behavioral comorbidities [24].

A retrospective analysis of data from a large German multicenter study showed synaptic vesicle glycoprotein 2A modulators have a favorable adverse event profile, with BRV showing fewer psychobehavioral adverse events than LEV [28]. In EXPERIENCE, the incidences of psychiatric, cognitive, and behavioral TEAEs were similar in patients with psychiatric comorbidity who switched from LEV to BRV compared with patients who switched from other ASMs to BRV; this finding was also observed in patients without psychiatric comorbidity who switched from LEV to BRV compared with patients who switched from other ASMs to BRV.

### Subgroup analyses by post-stroke epilepsy status

Few patients in EXPERIENCE had post-stroke epilepsy (*n* = 51), BTRE (*n* = 68), or TBIE (*n* = 49); therefore, analyses by epilepsy etiology should be interpreted with caution. Neurological, cardiovascular, and diabetic comorbidities were more common in patients with than without post-stroke epilepsy. Cardiovascular disease and diabetes are predisposing risk factors for stroke [29], and some neurological comorbidities seen in patients with post-stroke epilepsy may be a consequence of initial stroke [30]. The higher incidence of these comorbidities among patients with post-stroke epilepsy may also be due to the higher proportion of patients aged ≥ 65 years in this subgroup [31, 32]. The incidence of neurological comorbidities among patients with post-stroke epilepsy was low (69.0%), given that these patients have a history of stroke. This is likely related to differing reporting practices in the real world. In some cases, historical stroke may not have been recorded as a neurological comorbidity.

Patients with post-stroke epilepsy tended to achieve higher ≥ 50% seizure reduction, seizure freedom, and continuous seizure freedom at 12 months than patients without post-stroke epilepsy. These differences in effectiveness outcomes are likely due to the difference in baseline characteristics; patients with post-stroke epilepsy had a lower median number of prior ASMs and median seizure frequency/28 days than patients without post-stroke epilepsy. These differences may also be due to the underlying nature of post-stroke epilepsy; a hospital-based observational survey reported that focal epilepsy due to post-stroke brain abnormalities was associated with a higher proportion of seizure-free patients than focal epilepsy due to other brain abnormalities [33]. The percentage of patients that discontinued BRV was similar in patients with and without post-stroke epilepsy. The incidence of TEAEs at all time points was higher in patients with post-stroke epilepsy than without post-stroke epilepsy.

Since the EXPERIENCE analysis was undertaken, outcomes of a subgroup analysis of patients with post-stroke epilepsy from the retrospective observational BRIVAracetam add‑on First Italian netwoRk Study (BRIVAFIRST) [34] have been published. In EXPERIENCE, the percentage of patients with post-stroke epilepsy who achieved ≥ 50% seizure reduction at 12 months was higher than in BRIVAFIRST (50.0% vs 41.7%). Seizure freedom at 12 months (continuous seizure freedom) was lower in EXPERIENCE (13.8% vs 34.7%); however, BRIVAFIRST used a different definition for this outcome (no seizures within the previous 6 months), and this reported difference may be because any patients who discontinued BRV in EXPERIENCE were deemed not seizure free. Overall, 33.3% of patients with post-stroke epilepsy in EXPERIENCE discontinued BRV, compared with 13.3% in BRIVAFIRST. Differences in study design and patient baseline demographics may have contributed to any differences reported between the two studies. Data from international retrospective studies from outside of Europe were included in EXPERIENCE.

### Subgroup analyses by BTRE status

Management of BTRE is complex, due to the high incidence of drug resistance in these patients and the use of antineoplastic medication concomitantly with ASMs, which increases the risk of drug–drug adverse events [23]. To avoid interference with antineoplastic drugs, non–enzyme-inducing ASMs, such as LEV and lamotrigine, are recommended as first-line treatment for BTRE [23]. BRV is a non–enzyme-inducing ASM and therefore may be beneficial to patients with BTRE over other enzyme-inducing ASMs. Furthermore, an in vitro study by Rizzo et al. showed that BRV may possess antineoplastic activity on glioma cells, suggesting that BRV treatment may be beneficial to patients with glioma [35]. A retrospective multicenter study in Italy suggested BRV is an effective treatment option for reducing seizure frequency in patients with BTRE [36]. After a mean of 10 months, a significant reduction in mean monthly seizure frequency was reported, ≥ 50% seizure reduction was achieved in 18.1% of patients, and 60.6% of patients were seizure free.

In EXPERIENCE, patients with BTRE had a shorter duration of epilepsy (12.0 vs 17.8 years) and lower median number of prior ASMs than those without BTRE (3.0 vs 5.0); however, median seizure frequency at index was higher (5.3 vs 4.0). Patients with BTRE had similar ≥ 50% seizure reduction, seizure freedom, and continuous seizure freedom as patients without BTRE at all time points. However, retention on BRV was lower in patients with BTRE. Patients with BTRE more commonly discontinued BRV due to “tolerability” reasons and less commonly discontinued due to “lack of effectiveness” than patients without BTRE. This may reflect the poor underlying health status of patients with brain tumors. The incidence of TEAEs was similar in patients with and without BTRE.

### Subgroup analyses by TBIE status

To our knowledge, EXPERIENCE is the first study to investigate the effectiveness and tolerability of BRV in patients with TBIE. Effectiveness data for patients with TBIE should be interpreted with caution, due to the small numbers of patients assessed at each time point. ≥ 50% seizure reduction, seizure freedom, continuous seizure freedom, and retention were similar or tended to be higher in patients with TBIE compared with patients without TBIE at various time points. “Lack of effectiveness” was the most common reason for discontinuation among patients with TBIE and few patients discontinued due to “tolerability” reasons. This is likely related to the low incidence of TEAEs in patients with TBIE. With exception to cognitive and behavioral TEAEs at 3 months, there were no psychiatric, cognitive, or behavioral TEAEs reported by patients with TBIE.

### Strengths and limitations

Patient enrollment in EXPERIENCE begun at the date of BRV availability in each country. Therefore, patients may have initiated BRV during the post-launch phase which may have contributed to inclusion of a higher percentage of patients with drug-resistant epilepsy, as evidenced by baseline characteristics [18]. Strengths of EXPERIENCE include the rigorous approach used for seizure analyses (patients with missing data due to BRV discontinuation were deemed to be non-responders for ≥ 50% seizure reduction and not seizure free) and the use of a common data model, which enabled the pooling of patient cohorts from different countries and a variety of centers.

There are limitations to the EXPERIENCE pooled analysis [18]. For the subgroup analyses by psychiatric comorbidity, data on the types of psychiatric comorbidities were unavailable. Given the small number of patients with post-stroke epilepsy (*n* = 51), with BTRE (*n* = 68), and with TBIE (*n* = 49) data for subgroup analyses by etiology should be interpreted with caution. Analyses by epilepsy etiology were based on the patient’s etiology, as documented in each of the non-interventional studies. No data were available regarding the timing of epilepsy diagnosis following traumatic brain injury. Misclassification of etiologies cannot be excluded. For example, eight patients who were classified as “without BTRE” in the non-interventional studies had a documented etiology of tumor-related epilepsy (more than one etiology could be recorded). Despite these limitations, the large sample size of BRV 12-month clinical data provided by EXPERIENCE enabled the assessment of effectiveness and tolerability among key subpopulations of interest. These subgroup analyses add to the limited published real-world evidence data on patients on BRV with different comorbidities and different etiologies.

## Conclusions

The subgroup analyses of patients from a variety of real-world settings suggest that BRV as prescribed in the real world is effective and well tolerated among patients with CLD, patients with psychiatric comorbidity, patients with post-stroke epilepsy, patients with BTRE, and patients with TBIE. BRV treatment did not appear to exacerbate CLD or psychiatric comorbidity, as shown by the low incidences of psychiatric, cognitive, and behavioral TEAEs.

### Supplementary Information

Below is the link to the electronic supplementary material.

Supplementary file1 (PDF 874 KB)

## Data Availability

Data from non-interventional studies are outside of UCB Pharma’s data sharing policy and are unavailable for sharing.
