# Supplementary material for: Effectiveness and tolerability of brivaracetam in patients with epilepsy stratified by comorbidities and etiology in the real world: 12-month subgroup data from the international EXPERIENCE pooled analysis
Source: J Neurol. 2024 Mar 4;271(6):3169–85. doi: 10.1007/s00415-024-12253-z (PMC11136785; doi:10.1007/s00415-024-12253-z)

Supplementary material

Effectiveness and tolerability of brivaracetam in patients with epilepsy stratified by comorbidities and etiology in the real world: 12-month subgroup data from the international EXPERIENCE pooled analysis

Journal: *Journal of Neurology*

Jerzy P. Szaflarski[1] • Hervé Besson[2] • Wendyl D'Souza[3] • Edward Faught[4] • Pavel Klein[5] • Markus Reuber[6] • Felix Rosenow[7,8] • Javier Salas-Puig[9] • Victor Soto Insuga[10] • Bernhard J. Steinhoff[11] • Adam Strzelczyk[7,8] • Dimitrios Bourikas[12] • Tony Daniels[13] • Florin Floricel[14] • David Friesen[15] • Cédric Laloyaux[16] • Vicente Villanueva[17]

[1] University of Alabama at Birmingham (UAB) Heersink School of Medicine Department of Neurology and UAB Epilepsy Center, Birmingham, AL, USA
[2] UCB Pharma, Breda, Netherlands
[3] Department of Medicine, St Vincent's Hospital Melbourne, The University of Melbourne, Melbourne, VIC, Australia
[4] Emory Epilepsy Center, Atlanta, GA, USA
[5] Mid-Atlantic Epilepsy and Sleep Center, Bethesda, MD, USA
[6] The University of Sheffield, Sheffield, UK
[7] Epilepsy Center Frankfurt Rhine-Main, Department of Neurology, Goethe University Frankfurt, Frankfurt, Germany
[8] LOEWE Center for Personalized Translational Epilepsy Research (CePTER), Goethe University Frankfurt, Frankfurt, Germany
[9] Universitari Vall d'Hebron, Barcelona, Spain
[10] Pediatric Neurology, Hospital Universitario Infantil Niño Jesús, Madrid, Spain
[11] Kork Epilepsy Center, Kehl-Kork and Medical Faculty, University of Freiburg, Freiburg, Germany
[12] UCB Pharma, Alimos, Greece
[13] UCB Pharma, Morrisville, NC, USA
[14] UCB Pharma, Monheim am Rhein, Germany
[15] UCB Pharma, Slough, England, UK
[16] UCB Pharma, Brussels, Belgium
[17] Refractory Epilepsy Unit, Hospital Universitario y Politécnico La Fe, EpiCARE member, Valencia, Spain

Corresponding author: Jerzy P. Szaflarski, University of Alabama at Birmingham (UAB) Heersink School of Medicine Department of Neurology and UAB Epilepsy Center, Birmingham, AL, USA

e-mail address: jszaflarski@uabmc.edu

Supplementary Appendix S1 Patients with focal-onset seizures who were on a BRV dose of ≤ 200 mg/day used as add-on at index

Among patients with focal-onset seizures at baseline and a brivaracetam (BRV) dose of ≤ 200 mg/day used as add-on at index, baseline demographics and epilepsy characteristics in each of the comorbidity and etiology subgroups were similar to those observed in the wider subgroup populations (Table 1 and Supplementary Table S2).

Subgroup analyses by cognitive/learning disability (CLD) comorbidity showed that, during the whole study follow-up, 37.1% (*n* = 356) of patients with and 32.4% (*n* = 1074) of patients without CLD discontinued BRV. Effectiveness assessments at 12 months showed ≥ 50% seizure reduction was achieved in 36.9% and 38.6% of patients with and without CLD, respectively (modified full analysis set [mFAS]) (Supplementary Fig. S2a); seizure freedom was achieved in 8.0% and 17.4% (full analysis set [FAS]) (Supplementary Fig. S2b); continuous seizure freedom was achieved in 5.5% and 13.8% (Supplementary Fig. S2c); and BRV retention was achieved in 66.7% and 72.8% (Supplementary Fig. S2d). At 12 months, treatment-emergent adverse events (TEAEs) since prior visit were reported in 11.9% and 9.1% of patients with and without CLD, respectively (Supplementary Table S3).

Subgroup analyses by psychiatric comorbidity showed that, during the whole study follow-up, 31.3% (*n* = 531) of patients with and 34.7% (*n* = 885) of patients without psychiatric comorbidity discontinued BRV. At 12 months, ≥ 50% seizure reduction was achieved in 39.0% and 37.8% of patients with and without psychiatric comorbidity, respectively (mFAS) (Supplementary Fig. S2a); seizure freedom was achieved in 15.1% and 14.2% (FAS) (Supplementary Fig. S2b); continuous seizure freedom was achieved in 12.7% and 10.4% (Supplementary Fig. S2c); and BRV retention was achieved in 71.9% and 71.3% (Supplementary Fig. S2d). TEAEs since prior visit at 12 months were reported in 10.2% and 9.5% of patients with and without psychiatric comorbidity, respectively (Supplementary Table S3).

Subgroup analyses by post-stroke epilepsy status showed that, during the whole study follow-up, 34.7% (*n* = 49) of patients with and 33.5% (*n* = 1210) of patients without post-stroke epilepsy discontinued BRV. At 12 months, ≥ 50% seizure reduction was achieved in 39.1% and 38.0% of patients with and without post-stroke epilepsy, respectively (mFAS) (Supplementary Fig. S3a); seizure freedom was achieved in 34.4% and 14.9% (FAS)

(Supplementary Fig. S3b); continuous seizure freedom was achieved in 28.1% and 12.0% (Supplementary Fig. S3c); and BRV retention was achieved in 68.8% and 71.7% (Supplementary Fig. S3d). TEAEs since prior visit at 12 months were reported in 17.6% and 8.2% of patients with and without post-stroke epilepsy, respectively (Supplementary Table S4).

Subgroup analyses by brain tumor–related epilepsy (BTRE) status showed that, during the whole study follow-up, 39.0% (*n* = 59) of patients with BTRE and 33.3% (*n* = 1200) without BTRE discontinued BRV. At 12 months, ≥ 50% seizure reduction was achieved in 35.1% and 38.2% of patients with and without BTRE, respectively (mFAS) (Supplementary Fig. S3a); seizure freedom was achieved in 17.5% and 15.5% (FAS) (Supplementary Fig. S3b); continuous seizure freedom was achieved in 10.0% and 12.8% (Supplementary Fig. S3c); and BRV retention was achieved in 67.2% and 71.8% (Supplementary Fig. S3d). TEAEs since prior visit at 12 months were reported in 11.6% and 8.4% of patients with and without BTRE, respectively (Supplementary Table S4).

Subgroup analyses by traumatic brain injury–related epilepsy (TBIE) status showed that, during the whole study follow-up, 22.0% (*n* = 41) of patients with TBIE and 33.9% (*n* = 1218) of patients without TBIE discontinued BRV. At 12 months, ≥ 50% seizure reduction was achieved in 50.0% and 37.7% of patients with and without TBIE, respectively (mFAS) (Supplementary Fig. S3a); seizure freedom was achieved in 20.0% and 15.5% (FAS) (Supplementary Fig. S3b); continuous seizure freedom was achieved in 16.0% and 12.5% (Supplementary Fig. S3c); and BRV retention was achieved in 82.9% and 71.2% (Supplementary Fig. S3d). TEAEs since prior visit at 12 months were reported in 3.4% and 8.7% of patients with and without TBIE, respectively (Supplementary Table S4).

Supplementary Table S1 TEAEs since prior visit in patients with psychiatric comorbidity who switched from LEV to BRV and in patients who switched from other ASMs to BRV at index, and in patients without psychiatric comorbidity who switched from LEV to BRV and in patients who switched from other ASMs at index (FAS)

	3 months				6 months				12 months			
	With psychiatric comorbidity		Without psychiatric comorbidity		With psychiatric comorbidity		Without psychiatric comorbidity		With psychiatric comorbidity		Without psychiatric comorbidity	
**Patients, *n* (%)**	**Switched from LEV to BRV *n* = 225**	**Switched from other ASMs to BRV *n* = 317**	**Switched from LEV to BRV *n* = 441**	**Switched from other ASMs to BRV *n* = 486**	**Switched from LEV to BRV *n* = 189**	**Switched from other ASMs to BRV *n* = 273**	**Switched from LEV to BRV *n* = 404**	**Switched from other ASMs to BRV *n* = 444**	**Switched from LEV to BRV *n* = 153**	**Switched from other ASMs to BRV *n* = 241**	**Switched from LEV to BRV *n* = 368**	**Switched from other ASMs to BRV *n* = 408**
Any TEAEs	66 (29.3)	81 (25.6)	104 (23.6)	123 (25.3)	25 (13.2)	39 (14.3)	61 (15.1)	59 (13.3)	14 (9.2)	24 (10.0)	36 (9.8)	33 (8.1)
Severity of TEAEs[a,b]												
Mild	31 (53.4)[c]	23 (39.7)[c]	41 (55.4)[d]	33 (44.0)[e]	11 (50.0)[f]	18 (62.1)[g]	20 (54.1)[h]	12 (38.7)[i]	4 (30.8)[j]	13 (72.2)[k]	5 (23.8)[l]	10 (47.6)[l]
Moderate	21 (36.2)[c]	25 (43.1)[c]	22 (29.7)[d]	33 (44.0)[e]	8 (36.4)[f]	10 (34.5)[g]	14 (37.8)[h]	17 (54.8)[i]	7 (53.8)[j]	5 (27.8)[k]	16 (76.2)[l]	8 (38.1)[l]
Severe	6 (10.3)[c]	9 (15.5)[c]	11 (14.9)[d]	9 (12.0)[e]	3 (13.6)[f]	1 (3.4)[g]	3 (8.1)[h]	2 (6.5)[i]	2 (15.4)[j]	0	0	3 (14.3)[l]
Life-threatening	0	1 (1.7)[c,m]	0	0	0	0	0	0	0	0	0	0
Psychiatric TEAEs[n]	18 (8.0)	19 (6.0)	24 (5.4)	29 (6.0)	5 (2.6)	5 (1.8)	15 (3.7)	8 (1.8)	3 (2.0)	8 (3.3)	12 (3.3)	8 (2.0)
Cognitive TEAEs	4 (1.8)	11 (3.5)	9 (2.0)	15 (3.1)	1 (0.5)	4 (1.5)	5 (1.2)	8 (1.8)	0	4 (1.7)	4 (1.1)	3 (0.7)
Behavioral TEAEs	11 (4.9)	15 (4.7)	22 (5.0)	25 (5.1)	5 (2.6)	7 (2.3)	11 (2.7)	10 (2.3)	4 (2.6)	2 (0.8)	6 (1.6)	4 (1.0)
TEAEs[o,p] reported by ≥ 3% of patients at any time point												
Dizziness	13 (5.8)	16 (5.0)	16 (3.6)	19 (3.9)	2 (1.1)	4 (1.5)	4 (1.0)	6 (1.4)	3 (2.0)	4 (1.7)	2 (0.5)	2 (0.5)
Somnolence	11 (4.9)	10 (3.2)	18 (4.1)	19 (3.9)	6 (3.2)	8 (2.9)	12 (3.0)	8 (1.8)	6 (3.9)	5 (2.1)	10 (2.7)	5 (1.2)
Irritability	9 (4.0)	13 (4.1)	17 (3.9)	18 (3.7)	5 (2.6)	5 (1.8)	7 (1.7)	9 (2.0)	4 (2.6)	0	3 (0.8)	3 (0.7)
Fatigue	8 (3.6)	15 (4.7)	16 (3.6)	20 (4.1)	3 (1.6)	6 (2.2)	2 (0.5)	7 (1.6)	0	5 (2.1)	1 (0.3)	2 (0.5)
Depression	7 (3.1)	8 (2.5)	6 (1.4)	11 (2.3)	0	1 (0.4)	2 (0.5)	0	0	1 (0.4)	2 (0.5)	1 (0.2)

AE adverse event, *ASM* antiseizure medication, *BRV* brivaracetam, *FAS* full analysis set, *LEV* levetiracetam, *TEAE* treatment-emergent adverse event

[a]Patients with reported severity; [b]Excluding patients who had an AE that was not further described; [c]$n = 58$; [d]$n = 74$; [e]$n = 75$; [f]$n = 22$; [g]$n = 29$; [h]$n = 37$; [i]$n = 31$; [j]$n = 13$; [k]$n = 18$; [l]$n = 21$; [m]One TEAE of suicide was documented as life-threatening; [n]Behavioral TEAEs that fulfilled the criteria for psychiatric TEAEs were included in the psychiatric TEAEs category; [o]Medical Dictionary for Regulatory Activities version 24.1; [p]Patients with recorded AE that was not further described at 3 months/6 months/12 months: patients with psychiatric comorbidity and switched from LEV to BRV, 11 (4.9%)/5 (2.6%)/1 (0.7%); patients with psychiatric comorbidity and switched from other ASMs to BRV, 6 (1.9%)/5 (1.8%)/0; patients without psychiatric comorbidity and switched from LEV to BRV, 4 (0.9%)/15 (3.7%)/6 (1.6%); patients without psychiatric comorbidity and switched from other ASMs to BRV, 8 (1.6%)/14 (3.2%)/8 (2.0%)

Supplementary Table S2 Baseline demographics and epilepsy characteristics in patients with focal-onset seizures at baseline and a BRV dose of ≤ 200 mg/day used as add-on (FAS)

	CLD comorbidity		Psychiatric comorbidity		Post-stroke epilepsy status		BTRE status		TBIE status	
	**With CLD** ***N*** **= 356**	**Without CLD** ***N*** **= 1078**	**With psychiatric comorbidity** ***N*** **= 533**	**Without psychiatric comorbidity** ***N*** **= 887**	**With post-stroke epilepsy** ***N*** **= 49**	**Without post-stroke epilepsy** ***N*** **= 1214**	**With BTRE** ***N*** **= 59**	**Without BTRE** ***N*** **= 1204**	**With TBIE** ***N*** **= 42**	**Without TBIE** ***N*** **= 1221**
Age at baseline, *n* (%), years										
16–49	297 (83.4)	722 (67.0)	378 (70.9)	629 (15.4)	17 (34.7)	880 (72.5)	43 (72.9)	854 (70.9)	24 (57.1)	873 (71.5)
50–64	44 (12.4)	238 (22.1)	112 (21.0)	168 (18.9)	12 (24.5)	237 (19.5)	12 (20.3)	237 (19.7)	8 (19.0)	241 (19.7)
65–74	13 (3.7)	78 (7.2)	31 (5.8)	60 (6.8)	10 (20.4)	70 (5.8)	2 (3.4)	78 (6.5)	9 (21.4)	71 (5.8)
≥ 75	2 (0.6)	40 (3.7)	12 (2.3)	30 (3.4)	10 (20.4)	27 (2.2)	2 (3.4)	35 (2.9)	1 (2.4)	36 (2.9)
Sex, *n* (%)										
Male	189 (53.1)	502 (46.6)	246 (46.2)	438 (49.4)	26 (53.1)	576 (47.4)	32 (54.2)	570 (47.3)	29 (69.0)	573 (46.9)
Female	167 (46.9)	576 (53.4)	287 (53.8)	449 (50.6)	23 (46.9)	638 (52.6)	27 (45.8)	634 (52.7)	13 (31.0)	648 (53.1)
Duration of epilepsy, median (Q1, Q3), years	16.0 (4.0, 28.0)	18.0 (9.0, 31.0)[a]	18.0 (8.0, 31.0)[b]	17.0 (8.0, 30.0)[c]	23.5 (3.0, 55.0)[d]	17.0 (7.0, 29.5)[e]	13.0 (4.0, 28.0)	17.0 (8.0, 30.0)[f]	19.0 (9.0, 29.0)[g]	17.0 (7.0, 30.0)[h]
Seizure types at baseline,[i] *n* (%)										
Focal-onset	347 (97.5)	1075 (99.7)	527 (98.9)	881 (99.3)	49 (100.0)	1202 (99.0)	59 (100.0)	1192 (99.0)	41 (97.6)	1210 (99.1)
Focal-onset with secondary generalization	202 (78.0)[j]	429 (53.2)[k]	264 (67.5)[l]	362 (54.0)[m]	25 (69.4)[n]	556 (65.2)[o]	20 (55.6)[n]	561 (65.8)[o]	22 (73.3)[p]	559 (65.1)[q]
Generalized-onset	8 (2.2)	2 (0.2)	7 (1.3)	3 (0.3)	0	10 (0.8)	0	10 (0.8)	0	10 (0.8)
Unknown-onset	9 (2.5)	3 (0.3)	6 (1.1)	6 (0.7)	0	12 (1.0)	0	12 (1.0)	1 (2.4)	11 (0.9)
Seizure frequency/28 days at index, median (Q1, Q3)	7.7 (3.0, 30.0)[r]	4.0 (1.0, 12.0)[s]	4.0 (1.0, 12.0)[t]	4.3 (1.7, 13.3)[u]	1.0 (0.7, 6.0)[v]	4.0 (1.5, 13.0)[w]	6.0 (2.0, 12.0)[x]	4.0 (1.3, 12.0)[y]	2.3 (1.0, 6.0)	4.0 (1.3, 12.6)[z]
Most common etiology (≥ 5% of patients),[i,aa] *n* (%)										

Malformation of cortical development	71 (19.9)	187 (17.3)	105 (19.7)	151 (17.0)	0	234 (19.3)	1 (1.7)	233 (19.4)	1 (2.4)	233 (19.1)
Genetic	8 (2.2)	10 (0.9)	8 (1.5)	10 (1.1)	0	18 (1.5)	1 (1.7)	17 (1.4)	0	18 (1.5)
Tumor-related	11 (3.1)	62 (5.8)	19 (3.6)	52 (5.9)	1 (2.0)	62 (5.1)	55 (93.2)	8 (0.7)	0	63 (5.2)
Vascular	11 (3.1)	68 (6.3)	27 (5.1)	51 (5.7)	42 (85.7)	25 (2.1)	0	67 (5.6)	0	67 (5.5)
Traumatic	11 (3.1)	37 (3.4)	18 (3.4)	29 (3.3)	0	44 (3.6)	2 (3.4)	42 (3.5)	41 (97.6)	3 (0.2)
Post infectious	9 (2.5)	22 (2.0)	5 (0.9)	26 (2.9)	3 (6.1)	25 (2.1)	0	28 (2.3)	0	28 (2.3)
Most common comorbid conditions (≥ 10% of patients),[i] *n* (%)										
CLD	356 (100.0)	0	152 (28.6)[ab]	194 (22.0)[ac]	12 (24.5)	309 (25.6)[ad]	8 (13.6)	313 (26.1)[ae]	11 (26.2)	310 (25.5)[af]
Neurological	78 (41.5)[ag]	166 (23.3)[ah]	97 (31.2)[ai]	149 (25.3)[aj]	19 (67.9)[ak]	210 (30.2)[al]	5 (19.2)[am]	224 (32.1)[an]	15 (62.5)[ao]	214 (30.6)[ap]
Psychiatric	152 (43.9)[aq]	379 (35.5)[ar]	533 (100.0)	0	18 (36.7)	457 (38.3)[as]	17 (29.8)[at]	458 (38.6)[au]	16 (39.0)[av]	459 (38.2)[aw]
Cardiovascular disease	20 (7.0)[ax]	105 (11.9)[ay]	58 (13.2)[az]	67 (9.2)[ba]	25 (51.0)	100 (8.9)[bb]	3 (5.4)[bc]	122 (10.9)[bd]	7 (17.9)[be]	118 (10.4)[bf]
Diabetes/endocrine	14 (4.9)[ax]	38 (4.3)[bg]	24 (5.5)[az]	27 (3.7)[bh]	6 (12.2)	46 (4.1)[bi]	2 (3.6)[bc]	50 (4.5)[bj]	1 (2.6)[be]	51 (4.5)[bk]
Cancer	2 (1.3)[bl]	18 (3.2)[bm]	5 (2.0)[bn]	15 (3.2)[bo]	1 (3.6)[ak]	19 (2.7)[al]	8 (30.8)[am]	12 (1.7)[an]	1 (4.2)[ao]	19 (2.7)[ap]
Prior (lifetime) LEV	167 (62.1)[bp]	474 (61.6)[bq]	256 (62.0)[br]	378 (61.7)[bs]	21 (60.0)[bt]	476 (57.1)[bu]	23 (54.8)[g]	474 (57.4)[bv]	19 (61.3)[bw]	478 (57.1)[bx]
Switched from LEV or other ASMs to BRV, *n* (%)										
Switch from LEV	144 (41.3)[by]	475 (44.6)[bz]	220 (41.7)[ca]	396 (45.3)[cb]	25 (53.2)[v]	447 (37.4)[cc]	29 (50.0)[cd]	443 (37.4)[ce]	12 (29.3)[av]	460 (38.3)[aw]
Switch from other ASMs	203 (58.2)[by]	570 (53.6)[bz]	299 (56.7)[ca]	465 (53.2)[cb]	20 (42.6)[v]	730 (61.0)[cc]	27 (46.6)[cd]	723 (61.0)[ce]	27 (65.9)[av]	723 (60.1)[aw]
No switch	2 (0.6)[by]	19 (1.8)[bz]	8 (1.5)[ca]	13 (1.5)[cb]	2 (4.3)[v]	19 (1.6)[cc]	2 (3.4)[cd]	19 (1.6)[ce]	2 (4.9)[av]	19 (1.6)[aw]
Monotherapy/polytherapy at index, *n* (%)										
Monotherapy[cf]	0	0	0	0	0	0	0	0	0	0
Polytherapy[cg]	356 (100.0)	1078 (100.0)	533 (100.0)	887 (100.0)	49 (100.0)	1214 (100.0)	59 (100.0)	1204 (100.0)	42 (100.0)	1221 (100.0)
Number of prior ASMs at index,[ch] median (Q1, Q3)	7.0 (4.0, 10.0)[by]	4.0 (2.0, 7.0)[bz]	5.0 (3.0, 8.0)[ca]	5.0 (2.0, 8.0)[cb]	2.0 (1.0, 5.0)[v]	5.0 (2.0, 8.0)[cc]	3.0 (1.0, 6.0)[cd]	5.0 (2.0, 8.0)[ce]	4.0 (2.0, 7.0)[av]	5.0 (2.0, 8.0)[aw]
0–1, *n* (%)	34 (9.7)[by]	174 (16.4)[bz]	69 (13.1)[ca]	136 (15.6)[cb]	20 (42.6)[v]	184 (15.4)[cc]	17 (29.3)[cd]	187 (15.8)[ce]	5 (12.2)[av]	199 (16.6)[aw]
2–3, *n* (%)	39 (11.2)[by]	259 (24.3)[bz]	93 (17.6)[ca]	204 (23.3)[cb]	13 (27.7)[v]	257 (21.5)[cc]	17 (29.3)[cd]	253 (21.4)[ce]	13 (31.7)[av]	257 (21.4)[aw]

4–6, *n* (%)	96 (27.5)[by]	316 (29.7)[bz]	162 (30.7)[ca]	247 (28.3)[cb]	6 (12.8)[v]	343 (28.7)[cc]	14 (24.1)[cd]	335 (28.3)[ce]	10 (24.4)[av]	339 (28.2)[aw]
≥ 7, *n* (%)	180 (51.6)[by]	315 (29.6)[bz]	203 (38.5)[ca]	287 (32.8)[cb]	8 (17.0)[v]	412 (34.4)[cc]	10 (17.2)[cd]	410 (34.6)[ce]	13 (31.7)[av]	407 (33.9)[aw]
Number of concomitant maintenance ASMs at index, median (Q1, Q3)	3.0, (1.0, 4.0)	2.0 (1.0, 3.0)	2.0 (1.0, 3.0)	2.0 (1.0, 3.0)	1.0 (1.0, 2.0)	2.0 (1.0, 3.0)	2.0 (1.0, 3.0)	2.0 (1.0, 3.0)	2.0 (1.0, 3.0)	2.0 (1.0, 3.0)

ASM antiseizure medication, *BRV* brivaracetam, *BTRE* brain tumor–related epilepsy, *CLD* cognitive/learning disability, *FAS* full analysis set, *LEV* levetiracetam, *Q1* 25th quartile, *Q3* 75th quartile, *TBIE* traumatic brain injury–related epilepsy

[a]*n* = 1055; [b]*n* = 523; [c]*n* = 867; [d]*n* = 48; [e]*n* = 1184; [f]*n* = 1173; [g]*n* = 42; [h]*n* = 1190; [i]Patients could have had more than one response; [j]*n* = 259; [k]*n* = 806; [l]*n* = 391; [m]*n* = 670; [n]*n* = 36; [o]*n* = 853; [p]*n* = 30; [q]*n* = 859; [r]*n* = 308; [s]*n* = 902; [t]*n* = 456; [u]*n* = 740; [v]*n* = 47; [w]*n* = 1169; [x]*n* = 59; [y]*n* = 1157; [z]*n* = 1174; [aa]Patients with unknown or other etiology: patients with CLD, 239 (67.1%); patients without CLD, 693 (64.3%); patients with psychiatric comorbidity, 353 (66.2%); patients without psychiatric comorbidity, 571 (64.4%); patients with post-stroke epilepsy, 3 (6.1%); patients without post-stroke epilepsy, 811 (66.8%); patients without BTRE, 814 (67.6%); patients without TBIE, 814 (66.7%); [ab]*n* = 531; [ac]*n* =882; [ad]*n* = 1207; [ae]*n* = 1197; [af]*n* = 1214; [ag]*n* = 188; [ah]*n* = 712; [ai]*n* = 311; [aj]*n* = 590; [ak]*n* = 28; [al]*n* = 696; [am]*n* = 26; [an]*n* = 698; [ao]*n* = 24; [ap]*n* = 700; [aq]*n* = 346; [ar]*n* = 1067; [as]*n* = 1194; [at]*n* = 57; [au]*n* = 1186; [av]*n* = 41; [aw]*n* = 1202; [ax]*n* = 287; [ay]*n* = 879; [az]*n* = 439; [ba]*n* = 725; [bb]*n* = 1124; [bc]*n* = 56; [bd]*n* = 1117; [be]*n* = 39; [bf]*n* = 1134; [bg]*n* = 881; [bh]*n* = 727; [bi]*n* = 1126; [bj]*n* = 1119; [bk]*n* = 1136; [bl]*n* = 153; [bm]*n* = 569; [bn]*n* = 253; [bo]*n* = 471; [bp]*n* = 269; [bq]*n* = 769; [br]*n* = 413; [bs]*n* = 613; [bt]*n* = 35; [bu]*n* = 833; [bv]*n* = 826; [bw]*n* = 31; [bx]*n* = 837; [by]*n* = 349; [bz]*n* = 1064; [ca]*n* = 527; [cb]*n* = 874; [cc]*n* = 1196; [cd]*n* = 58; [ce]*n* = 1185; [cf]No concomitant ASM at index; [cg]Concomitant ASM(s) at index; [ch]Any ASM used and stopped before BRV initiation

Supplementary Table S3 TEAEs since prior visit, by comorbidity, in patients with focal-onset seizures and a BRV dose of ≤ 200 mg/day used as add-on at index

	**3 months**				**6 months**				**12 months**			
	**CLD comorbidity**		**Psychiatric comorbidity**		**CLD comorbidity**		**Psychiatric comorbidity**		**CLD comorbidity**		**Psychiatric comorbidity**	
**Patients, *n* (%)**	**With CLD *n* = 329**	**Without CLD *n* = 1026**	**With psychiatric comorbidity *n* = 497**	**Without psychiatric comorbidity *n* = 844**	**With CLD *n* = 283**	**Without CLD *n* = 913**	**With psychiatric comorbidity *n* = 421**	**Without psychiatric comorbidity *n* = 765**	**With CLD *n* = 244**	**Without CLD *n* = 822**	**With psychiatric comorbidity *n* = 361**	**Without psychiatric comorbidity *n* = 698**
Any TEAEs	76 (23.1)	280 (27.3)	134 (27.0)	217 (25.7)	46 (16.3)	136 (14.9)	63 (15.0)	115 (15.0)	29 (11.9)	75 (9.1)	37 (10.2)	66 (9.5)
Severity of TEAEs[a,b]												
Mild	29 (52.7)[c]	100 (48.8)[d]	53 (48.2)[e]	72 (50.0)[f]	12 (38.7)[g]	55 (60.4)[h]	31 (59.6)[i]	32 (48.5)[j]	8 (36.4)[k]	25 (49.0)[l]	17 (56.7)[m]	15 (35.7)[n]
Moderate	18 (32.7)[c]	78 (38.0)[d]	41 (37.3)[e]	54 (37.5)[f]	16 (51.6)[g]	33 (36.3)[h]	18 (34.6)[i]	31 (47.0)[j]	11 (50.0)[k]	24 (47.1)[l]	11 (36.7)[m]	24 (57.1)[n]
Severe	8 (14.5)[c]	26 (12.7)[d]	15 (13.6)[e]	18 (12.5)[f]	3 (9.7)[g]	3 (3.3)[h]	3 (5.8)[i]	3 (4.5)[j]	3 (13.6)[k]	2 (3.9)[l]	2 (6.7)[m]	3 (7.1)[n]
Life-threatening	0	1 (0.5)[d,o]	1 (0.9)[e,o]	0	0	0	0	0	0	0	0	0
Psychiatric TEAEs[p]	18 (5.5)	70 (6.8)	36 (7.2)	48 (5.7)	11 (3.9)	23 (2.5)	10 (2.4)	22 (2.9)	9 (3.7)	20 (2.4)	10 (2.8)	19 (2.7)
Cognitive TEAEs	7 (2.1)	29 (2.8)	12 (2.4)	22 (2.6)	5 (1.8)	8 (0.9)	3 (0.7)	11 (1.4)	3 (1.2)	6 (0.7)	5 (1.4)	5 (0.7)
Behavioral TEAEs	25 (7.6)	51 (5.0)	25 (5.0)	48 (5.7)	16 (5.6)	20 (2.2)	14 (3.3)	19 (2.5)	8 (3.3)	8 (1.0)	6 (1.7)	9 (1.3)
TEAE[q,r],reported by ≥ 3% of patients at any time point												
Irritability	19 (5.8)	41 (4.0)	22 (4.4)	37 (4.4)	11 (3.9)	17 (1.9)	12 (2.9)	15 (2.0)	4 (1.6)	7 (0.9)	5 (1.4)	6 (0.9)
Somnolence	13 (4.0)	46 (4.5)	21 (4.2)	38 (4.5)	9 (3.2)	26 (2.8)	14 (3.3)	20 (2.6)	6 (2.5)	20 (2.4)	10 (2.8)	15 (2.1)
Fatigue	6 (1.8)	51 (5.0)	24 (4.8)	32 (3.8)	1 (0.4)	19 (2.1)	9 (2.1)	9 (1.2)	2 (0.8)	8 (1.0)	6 (1.7)	3 (0.4)
Dizziness	8 (2.4)	53 (5.2)	28 (5.6)	34 (4.0)	1 (0.4)	15 (1.6)	6 (1.4)	10 (1.3)	0	9 (1.1)	5 (1.4)	4 (0.6)

AE adverse event, *BRV* brivaracetam, *CLD* cognitive/learning disability, *TEAE* treatment-emergent adverse event

[a]Patients with reported severity; [b]Excluding patients who had an AE that was not further described; [c]*n* = 55; [d]*n* = 205; [e]*n* = 110; [f]*n* = 144; [g]*n* = 31; [h]*n* = 91;[i]*n* = 52; [j]*n* = 66; [k]*n* = 22; [l]*n* = 51; [m]*n* = 30; [n]*n* = 42; [o]One TEAE of suicide was documented as life threatening; [p]Behavioral TEAEs that fulfilled the criteria for psychiatric TEAEs were included in the psychiatric TEAEs category; [q]Medical Dictionary for Regulatory Activities version 24.1; [r]Patients with recorded AE that was not further described at 3 months/6 months/12 months: patients with CLD, 6 (1.8%)/12 (4.2%)/6 (2.5%); patients without CLD, 18 (1.8%)/24 (2.6%)/10 (1.2%); patients with psychiatric comorbidity, 12 (2.4%)/8 (1.9%)/1 (0.3%); patients without psychiatric comorbidity, 12 (1.4%)/28 (3.7%)/15 (2.1%)

Supplementary Table S4 TEAEs since prior visit, by etiology, in patients with focal-onset seizures and a BRV dose of ≤ 200 mg/day used as add-on at index

	**3 months**						**6 months**						**12 months**					
	**Post-stroke epilepsy status**		**BTRE status**		**TBIE status**		**Post-stroke epilepsy status**		**BTRE status**		**TBIE status**		**Post-stroke epilepsy status**		**BTRE status**		**TBIE status**	
**Patients, *n* (%)**	**With post-stroke epilepsy *n* = 42**	**Without post-stroke epilepsy *n* = 1142**	**With BTRE *n* = 57**	**Without BTRE *n* = 1127**	**With TBIE *n* = 38**	**Without TBIE *n* = 1146**	**With post-stroke epilepsy *n* = 37**	**Without post-stroke epilepsy *n* = 1020**	**With BTRE *n* = 47**	**Without BTRE *n* = 1010**	**With TBIE *n* = 33**	**Without TBIE *n* = 1024**	**With post-stroke epilepsy *n* = 34**	**Without post-stroke epilepsy *n* = 913**	**With BTRE *n* = 43**	**Without BTRE *n* = 904**	**With TBIE *n* = 29**	**Without TBIE *n* = 918**
Any TEAEs	15 (35.7)	281 (24.6)	16 (28.1)	280 (24.8)	7 (18.4)	289 (25.2)	10 (27.0)	145 (14.2)	7 (14.9)	148 (14.7)	2 (6.1)	153 (14.9)	6 (17.6)	75 (8.2)	5 (11.6)	76 (8.4)	1 (3.4)	80 (8.7)
Severity of TEAEs[a,b]																		
Mild	7 (77.8)[c]	103 (53.9)[d]	7 (63.6)[e]	103 (54.5)[f]	3 (60.0)[g]	107 (54.9)[h]	5 (71.4)[i]	53 (58.9)[j]	3 (100.0)[k]	55 (58.5)[l]	2 (100.0)[m]	56 (58.9)[n]	4 (100.0)[o]	22 (43.1)[p]	1(100.0)[q]	25 (46.3)[r]	1 (100.0)[s]	25 (46.3)[r]
Moderate	2 (22.2)[c]	69 (36.1)[d]	4 (36.4)[e]	67 (35.4)[f]	2 (40.0)[g]	69 (35.4)[h]	2 (28.6)[i]	35 (38.9)[j]	0	37 (39.4)[l]	0	37 (38.9)[n]	0	26 (51.0)[p]	0	26 (48.1)[r]	0	26 (48.1)[r]
Severe	0	18 (9.4)[d]	0	18 (9.5)[f]	0	18 (9.2)[h]	0	2 (2.2)[j]	0	2 (2.1)[l]	0	2 (2.1)[n]	0	3 (5.9)[p]	0	3 (5.6)[r]	0	3 (5.6)[r]
Life-threatening	0	1 (0.1)[d,t]	0	1 (0.5)[f,t]	0	1 (0.5)[h,t]	0	0	0	0	0	0	0	0	0	0	0	0
Psychiatric TEAEs[u]	6 (14.3)	67 (5.9)	5 (8.8)	68 (6.0)	0	73 (6.4)	1 (2.7)	26 (2.5)	3 (6.4)	24 (2.4)	0	27 (2.6)	1 (2.9)	23 (2.5)	1 (2.3)	23 (2.5)	0	24 (2.6)
Cognitive TEAEs	1 (2.4)	35 (3.1)	2 (3.5)	34 (3.0)	1 (2.6)	35 (3.1)	0	14 (1.4)	1 (2.1)	13 (1.3)	0	14 (1.4)	0	10 (1.1)	1 (2.3)	9 (1.0)	0	10 (1.1)
Behavioral TEAEs	4 (9.5)	56 (4.9)	3 (5.3)	57 (5.1)	2 (5.3)	58 (5.1)	1 (2.7)	27 (2.6)	1 (2.1)	27 (2.7)	0	28 (2.7)	0	10 (1.1)	0	10 (1.1)	0	10 (1.1)
TEAEs[v,w] reported by ≥ 3% of patients at any time point																		
Depression	3 (7.1)	27 (2.4)	3 (5.3)	27 (2.4)	0	30 (2.6)	0	3 (0.3)	0	3 (0.3)	0	3 (0.3)	1 (2.9)	3 (0.3)	0	4 (0.4)	0	4 (0.4)
Aggression	2 (4.8)	16 (1.4)	2 (3.5)	16 (1.4)	0	18 (1.6)	0	10 (1.0)	1 (2.1)	9 (0.9)	0	10 (1.0)	0	5 (0.5)	0	5 (0.6)	0	5 (0.5)
Fatigue	2 (4.8)	51 (4.5)	5 (8.8)	48 (4.3)	1 (2.6)	52 (4.5)	1 (2.7)	15 (1.5)	1 (2.1)	15 (1.5)	0	16 (1.6)	1 (2.9)	8 (0.9)	1 (2.3)	8 (0.9)	0	9 (1.0)
Somnolence	2 (4.8)	38 (3.3)	1 (1.8)	39 (3.5)	0	40 (3.5)	3 (8.1)	23 (2.3)	0	26 (2.6)	0	26 (2.5)	1 (2.9)	15 (1.6)	0	16 (1.8)	0	16 (1.7)
Irritability	1 (2.4)	43 (3.8)	2 (3.5)	42 (3.7)	2 (5.3)	42 (3.7)	0	19 (1.9)	0	20 (2.0)	0	20 (2.0)	0	5 (0.5)	0	5 (0.6)	0	5 (0.5)
Dizziness	0	47 (4.1)	2 (3.5)	45 (4.0)	1 (2.6)	46 (4.0)	0	12 (1.2)	1 (2.1)	11 (1.1)	0	12 (1.2)	0	4 (0.4)	0	4 (0.4)	0	4 (0.4)

AE adverse event, *BRV* brivaracetam, *BTRE* brain tumor–related epilepsy, *TBIE* traumatic brain injury–related epilepsy, *TEAE* treatment-emergent adverse event

[a]Patients with reported severity; [b]Excluding patients who had an AE that was not further described; [c]*n* = 9; [d]*n* = 191; [e]*n* = 11; [f]*n* = 189; [g]*n* = 5; [h]*n* = 195;[i]*n* = 7; [j]*n* = 90; [k]*n* = 3; [l]*n* = 94;[m]*n* = 2; [n]*n* = 95;[o]*n* = 4; [p]*n* = 51;[q]*n* = 1; [r]*n* = 54; [s]*n* = 1; [t]One TEAE of suicide was documented as life threatening; [u]Behavioral TEAEs that fulfilled the criteria for psychiatric TEAEs were included in the psychiatric TEAEs category; [v]Medical Dictionary for Regulatory Activities version 24.1; [w]Patients with recorded AE that was not further described at 3 months/6 months/12 months: patients with post-stroke epilepsy, 3 (7.1%)/2 (5.4%)/1 (2.9%); patients without post-stroke epilepsy, 21 (1.8%)/34 (3.3%)/15 (1.6%); patients with BTRE, 1 (1.8%)/1 (2.1%)/2 (4.7%); patients without BTRE, 23 (2.0%)/35 (3.5%)/14 (1.5%); patients with TBIE, 2 (5.3%)/1 (3.0%)/1 (3.4%); patients without TBIE, 22 (1.9%)/35 (3.4%)/15 (1.6%)

Supplementary Fig. S1 Analyses of effectiveness for patients with psychiatric comorbidity who switched from LEV to BRV and who switched from other ASMs to BRV at index, and in patients without psychiatric comorbidity who switched from LEV to BRV and who switched from other ASMs to BRV at index: (a) ≥ 50% seizure reduction (mFAS), (b) seizure freedom (FAS), (c) continuous seizure freedom (FAS), and (d) BRV retention (FAS). *n* represents the number of patients with data for the reported variable at each visit. Patients with missing data were excluded from all seizure analyses. Patients with missing data after BRV discontinuation were considered non responders and not seizure free. *ASM* antiseizure medication, *BRV* brivaracetam, *FAS* full analysis set, *LEV* levetiracetam, *mFAS* modified full analysis set

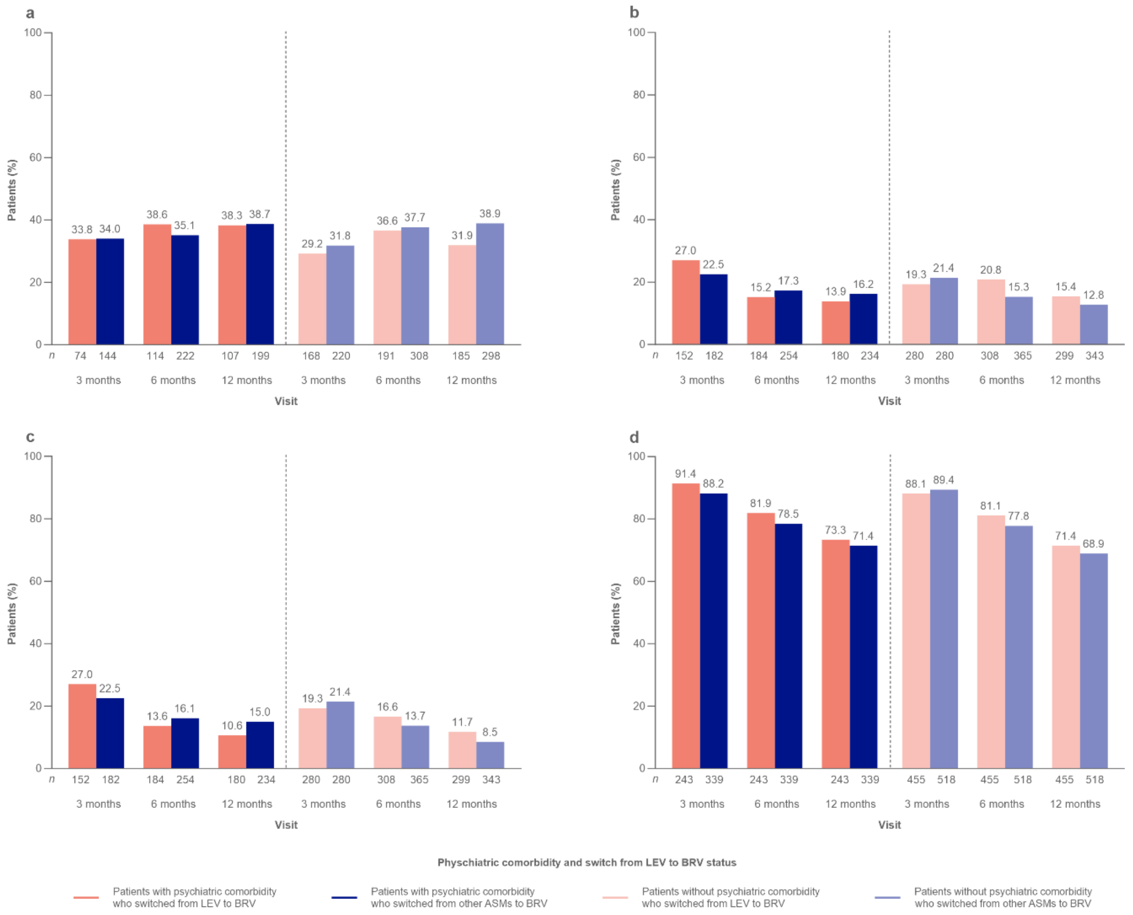

Supplementary Fig. S2 Analyses of effectiveness for patients with focal-onset seizures and a BRV dose of ≤ 200 mg/day used as add-on at index, by comorbidity (patients with/without CLD and patients with/without psychiatric comorbidity) at baseline: (**a**) ≥ 50% seizure reduction (mFAS), (**b**) seizure freedom (FAS), (**c**) continuous seizure freedom (FAS), and (**d**) BRV retention (FAS). *n* represents the number of patients with data for the reported variable at each visit. Patients with missing data were excluded from all seizure analyses. Patients with missing data after BRV discontinuation were considered non-responders and not seizure free. *BRV* brivaracetam, *CLD* cognitive/learning disability, *FAS* full analysis set, *mFAS* modified full analysis set

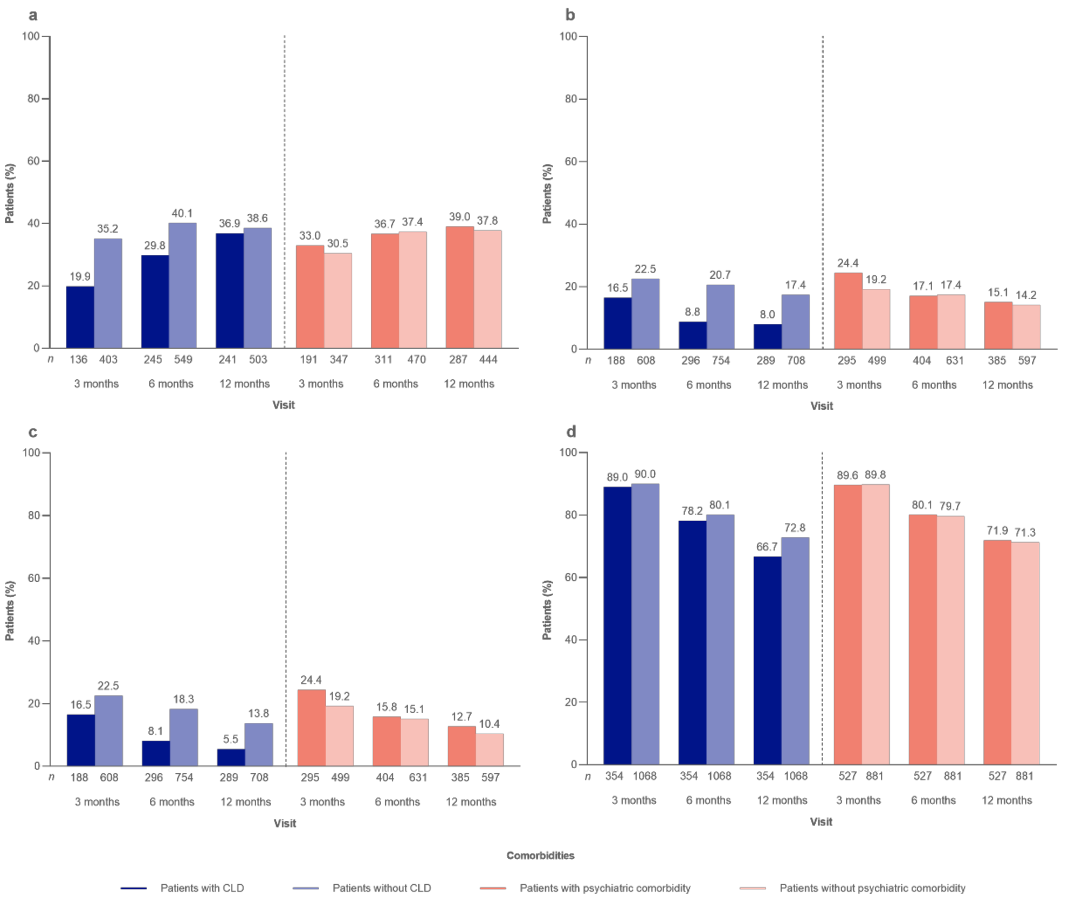

Supplementary Fig. S3 Analyses of effectiveness for patients with focal-onset seizures and a BRV dose of ≤ 200 mg/day used as add-on at index, by etiology (patients with/without post-stroke epilepsy, patients with/without BTRE, and patients with/without TBIE) at baseline: (**a**) ≥ 50% seizure reduction (mFAS), (**b**) seizure freedom (FAS), (**c**) continuous seizure freedom (FAS), and (**d**) BRV retention (FAS). *n* represents the number of patients with data for the reported variable at each visit. Patients with missing data were excluded from all seizure analyses. Patients with missing data after BRV discontinuation were considered non-responders and not seizure free. *BRV* brivaracetam, *BTRE* brain tumor–related epilepsy, *FAS* full analysis set, *mFAS* modified full analysis set, *TBIE* traumatic brain injury–related epilepsy

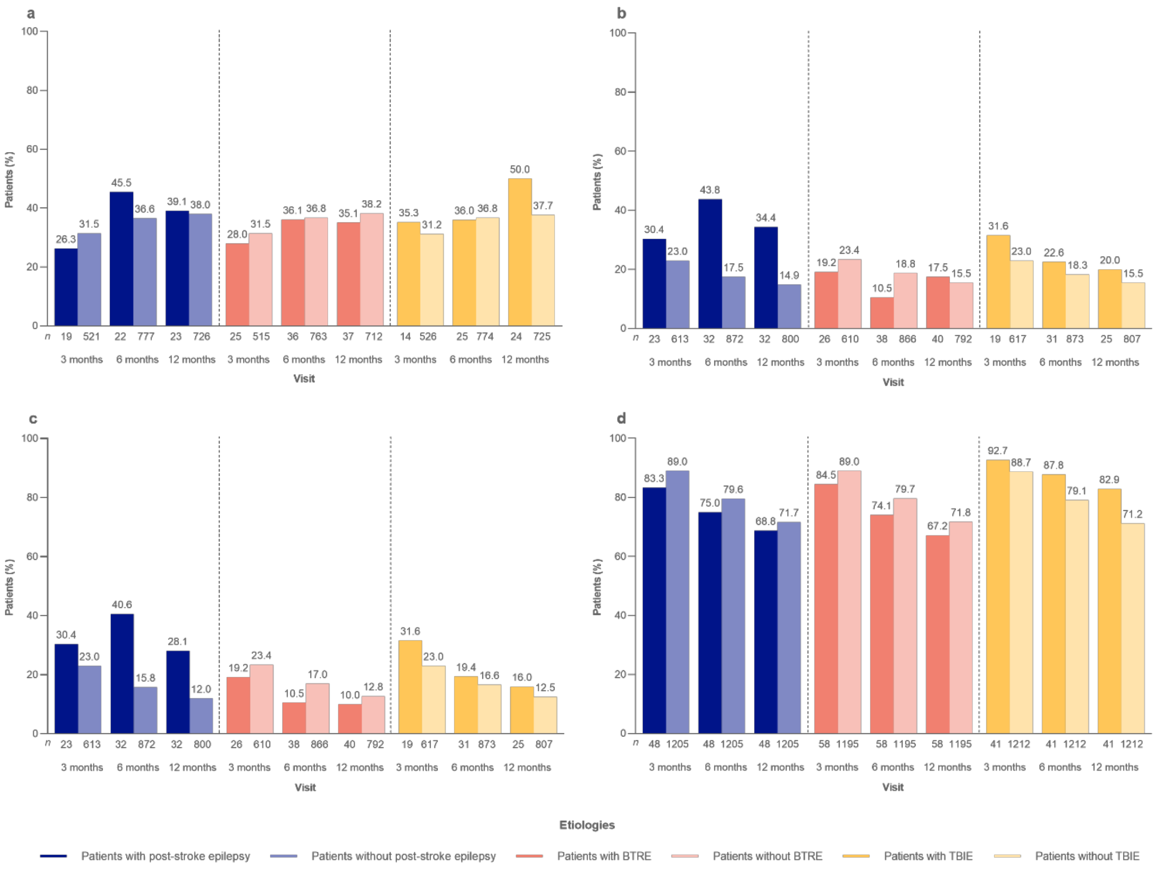

Supplement: Supplementary file 1 — Supplementary file1 (PDF 874 KB) [file 415_2024_12253_MOESM1_ESM.pdf]
